# Expression GWAS of *PGIP1* Identifies STOP1-Dependent and STOP1-Independent Regulation of *PGIP1* in Aluminum Stress Signaling in *Arabidopsis*

**DOI:** 10.3389/fpls.2021.774687

**Published:** 2021-12-17

**Authors:** Raj Kishan Agrahari, Takuo Enomoto, Hiroki Ito, Yuki Nakano, Emiko Yanase, Toshihiro Watanabe, Ayan Sadhukhan, Satoshi Iuchi, Masatomo Kobayashi, Sanjib Kumar Panda, Yoshiharu Y. Yamamoto, Hiroyuki Koyama, Yuriko Kobayashi

**Affiliations:** ^1^Faculty of Applied Biological Sciences, Gifu University, Gifu, Japan; ^2^Research Faculty of Agriculture, Hokkaido University, Hokkaido, Japan; ^3^Department of Biotechnology, Koneru Lakshmaiah Education Foundation, Guntur, India; ^4^Experimental Plant Division, RIKEN BioResource Research Center, Tsukuba, Japan; ^5^Department of Biochemistry, Central University of Rajasthan, Ajmer, India

**Keywords:** aluminum stress, *Arabidopsis thaliana*, GWAS, *PGIP1*, phosphoinositide signaling, transcription factor, NO signaling, STOP1

## Abstract

To elucidate the unknown regulatory mechanisms involved in aluminum (Al)-induced expression of *POLYGALACTURONASE-INHIBITING PROTEIN 1* (*PGIP1*), which is one of the downstream genes of SENSITIVE TO PROTON RHIZOTOXICITY 1 (STOP1) regulating Al-tolerance genes, we conducted a genome-wide association analysis of gene expression levels (eGWAS) of *PGIP1* in the shoots under Al stress using 83 *Arabidopsis thaliana* accessions. The eGWAS, conducted through a mixed linear model, revealed 17 suggestive SNPs across the genome having the association with the expression level variation in *PGIP1*. The GWAS-detected SNPs were directly located inside transcription factors and other genes involved in stress signaling, which were expressed in response to Al. These candidate genes carried different expression level and amino acid polymorphisms. Among them, three genes encoding NAC domain-containing protein 27 (NAC027), TRX superfamily protein, and R-R-type MYB protein were associated with the suppression of *PGIP1* expression in their mutants, and accordingly, the system affected Al tolerance. We also found the involvement of Al-induced endogenous nitric oxide (NO) signaling, which induces *NAC027* and *R-R-type MYB* genes to regulate *PGIP1* expression. In this study, we provide genetic evidence that STOP1-independent NO signaling pathway and STOP1-dependent regulation in phosphoinositide (PI) signaling pathway are involved in the regulation of *PGIP1* expression under Al stress.

## Introduction

In the last few decades, extensive studies in molecular physiological research for aluminum (Al) toxicity in acid soils (pH < 5.5) have found that activation of Al-tolerance genes governs Al resistance in plants (Delhaize and Ryan, 1995; Liu et al., 2014; Kochian et al., 2015). Several transcription factors that activate the transcription of critical Al-resistant genes (e.g., *ALUMINUM-ACTIVATED MALATE TRANSPORTER 1 (ALMT1)*; Tokizawa et al., 2015) have been identified, including SENSITIVE TO PROTON RHIZOTOXICITY 1 (STOP1; Iuchi et al., 2007). Al-inducible expression of STOP1-regulated genes plays critical roles in Al tolerance in *Arabidopsis* (Sawaki et al., 2009) and is conserved in various plant species (Ohyama et al., 2013). It is important to identify the mechanisms regulating gene expression related to Al tolerance, which would be helpful in the field of breeding and the management of crops in acid soil.

Several mechanisms regulating the expression of STOP1-regulated Al-tolerance genes have been reported. For example, the expression of *AtALMT1* under Al stress involves calcium signaling that includes CALCINEURIN B-LIKE PROTEIN 1 (Ligaba-Osena et al., 2017) and CALMODULIN-BINDING TRANSCRIPTION ACTIVATOR 2 (Tokizawa et al., 2015), and phosphatidylinositol signaling that includes PHOSPHATIDYLINOSITOL 4-KINASE (Wu et al., 2019). In addition, WRKY DNA-BINDING PROTEIN 46 suppresses *AtALMT1* (Ding et al., 2013). These regulators have been characterized in most Al-stress root responses. However, long-term stress leads to high accumulation of Al in the shoot, which is also directly related to shoot growth inhibition (Larsen et al., 1997, 2005; Sadhukhan et al., 2020). Although most of the Al signaling mechanism in shoot is unknown, for example recently we have found Al-inducible expression of *ALUMINUM SENSITIVE 3* [*ALS3*; encodes a bacterial-type ABC transporter-like protein that is involved in the translocation of Al (Larsen et al., 2005)] is STOP1-dependent and shows a specific response to Al in the shoots of *Arabidopsis* (Sawaki et al., 2016). By contrast, Al-inducible expression of *ALS3* in the shoots is dependent on similar signaling mechanisms in the roots, including phosphatidylinositol signaling, although the genes involved in the pathway differ between the shoots and roots (Wu et al., 2019; Sadhukhan et al., 2020). These observations suggest that understanding the regulatory mechanisms of gene expression in shoots is critically important to explore the complexity of the Al signaling pathway.

*POLYGALACTURONASE-INHIBITING PROTEIN 1* (*PGIP1*) gene expression is regulated by STOP1 and is strongly induced in the shoot along with *ALS*3 by mineral stress (especially Al) and acidic soil conditions (Sawaki et al., 2016). Although the contribution of PGIP1 to Al tolerance has not been studied yet, it has been speculated that PGIP1 plays a role in stabilizing the pectin in the cell wall under acidic conditions (Sawaki et al., 2009; Kobayashi et al., 2014). Al binds preferentially to unmethylated pectin, catalyzed by pectin methylesterase *via* nitric oxide (NO) signaling (Sun et al., 2016), which negatively affects cell wall structure and function by increasing rigidity and reducing cell expansion and mechanical extensibility, thus inhibiting plant growth (Tabuchi and Matsumoto, 2001; Sun et al., 2016). In contrast to *ALS3*, which is specifically expressed in Al, *PGIP1* is also responsive to abiotic stress. Specifically, *PGIP1* is induced by oligogalacturonides, a known degradation product of the cell wall in plant defense (Ferrari et al., 2003; Davidsson et al., 2017). This suggests that analysis of the response of *PGIP1* expression will provide an opportunity to study Al signaling pathways in the shoot that may reveal the cross talk between stress signaling pathways, including Al stress, when compared to previous studies of *ALS3* (Sadhukhan et al., 2020).

GWAS on the expression level difference of an Al-response gene is a powerful tool to identify the unknown upstream signaling pathways regulating the gene of interest (Atwell et al., 2010; Wang et al., 2020). GO enrichment and gene co-expression network analyses can add to the power of eGWAS in identifying functional candidate genes (Kobayashi et al., 2016; Sadhukhan et al., 2020; Song et al., 2021). We have conducted a genome-wide association study targeting gene expression level (eGWAS) that identified *cis*-mutations in the promoters of *NOD26-like intrinsic protein 1*; *1* (*NIP1*; *1*), which regulates hydrogen peroxide sensitivity, and *AtALMT1*; *multidrug and toxic compound extrusion* (*MATE*), which encodes an Al-responsive citrate transporter, is the determinant of the expression levels of these genes in roots (Sadhukhan et al., 2017; Nakano et al., 2020a, 2020b). In addition to the cis-locus, the eGWAS of *AtMATE* also revealed trans-loci associated with gene expression (Nakano et al., 2020b). Through eGWAS, we also found the involvement of phosphatidylinositol and calcium signaling in the regulation of *ALS3* expression under Al stress in *Arabidopsis* shoots (Sadhukhan et al., 2020). To identify the factors involved in Al signaling related to *PGIP1* expression, we conducted an eGWAS based on the expression level of *PGIP1* in *Arabidopsis thaliana* accessions with a reverse genetics approach. We propose both STOP1-dependent and STOP1-independent Al signalings for the transcriptional regulation of *PGIP1* in the shoots of *A. thaliana*.

## Materials and Methods

### Plant Materials

Seeds of 83 worldwide natural *A. thaliana* accessions (Atwell et al., 2010; Cao et al., 2011; Horton et al., 2012) used in our previous GWAS (Sadhukhan et al., 2020; Nakano et al., 2020b) and T-DNA insertion lines were obtained from the Arabidopsis Biological Resource Center (ABRC, Columbus, OH, United States), the Nottingham *Arabidopsis* Stock Centre (NASC, Nottingham, United Kingdom), and the RIKEN BioResource Center (RIKEN BRC, Tsukuba, Japan). Prior to experimental use, the procured seeds were multiplied by a single-seed descent process. Homozygosity was confirmed in T-DNA insertion mutant line using primers from the SALK database, following their protocols.^1^ The sequences of the primers are given in Supplementary Table S1. The T-DNA insertion mutants used in this study were *pgip1* (SALK_001662), *STOP1-*KO (SALK_114108), *at1g64105* (SAIL_235_D03), *at2g21950* (GK-707C08), *at3g24480* (GABI_017A08), *at5g15300* (SALK_044494C), *at5g38900* (SAIL_453_G03), *at5g43460* (SALK_067877C), *at5g58900* (SALK_084867C), *at5g58910* (SALK_064093C), *at1g51070* (SALK_104253C), *at2g38090* (SALK_127250C), *at2g04780* (SALK_113729C) and *plc9* (SALK_021982C).

### Plant Growth Conditions and Stress Treatment

Seedlings were grown on nylon mesh floating on modified MGRL solution (Fujiwara et al., 1992; 2% solution with 200 μM CaCl_2_; initial pH 5.6) for 10 days at 22°C using a 12-h photoperiod with 37 μMol m^−2^ s^−1^ photon flux density. The culture solution was renewed every 2 days. After 10 days, the seedlings were transferred to another modified MGRL solution (without P and pH 5.0) containing 25 μM AlCl_3_·6H_2_O (Sawaki et al., 2016). The shoots were harvested after 24 h of Al treatment and immediately frozen with liquid nitrogen for RNA extraction. The same Al toxic solution containing 50 μM 2-(4-carboxyphenyl)-4,4,5,5-tetramethyl-imidazoline-1-oxyl-3-oxide (cPTIO), an NO scavenger, was used to evaluate the effect of NO (D’Alessandro et al., 2013; Sun et al., 2016). The concentration of cPTIO was based on preliminary experiments from which maximum suppressed responses were obtained without affecting the plant root (Supplementary Figures S1, S2).

Soil culture was conducted using commercial acidic soil (PROTOLEAF, Tokyo, Japan; pH 4.2; 1:2.5 w/v soil/water solution; Agrahari et al., 2020). The acidic soil was neutralized by the addition of CaCO_3_ (Koyama et al., 2000; Sawaki et al., 2016; Agrahari et al., 2020; 4.0 g kg^−1^; pH 5.1; 1:2.5 w/v soil/water solution) and used as the control soil. Plants (100 seeds) were grown for 2 weeks at 22°C during a 12-h photoperiod with 37 μMol m^−2^ s^−1^. Throughout the experiment, the plants were irrigated daily with deionized water to maintain soil moisture. The soil pH (water) and exchangeable Al were determined using the method described by Koyama et al. (2000).

### RNA Extraction and Real-Time Quantitative Reverse Transcription PCR

Total RNA was isolated from the shoots using Sepasol-RNA I Super G (Nacalai Tesque, Kyoto, Japan) according to the manufacturer’s instructions. The RNA quality was analyzed using the A260/A280 ratio on a NanoVue Plus spectrophotometer (Biochrom, Holliston, United States). Total RNA was reverse-transcribed using ReverTra Ace quantitative PCR master mix with genomic DNA remover (Toyobo, Osaka, Japan) following the manufacturer’s instructions. The gene expression levels were quantified using SYBR Premix Ex Taq II (Takara Bio, Otsu, Japan) with a Dice Real Time System II MRQ thermal cycler (Takara Bio, Otsu, Japan) according to the manufacturer’s instructions. Briefly, all quantifications were carried out based on the real-time quantitative reverse transcription PCR (qRT-PCR) standard curve method of Bustin et al. (2009), as described by Kobayashi et al. (2014). For all quantifications, a standard curve was constructed using a cDNA dilution series, and the transcript levels of selected genes were quantified relative to that of the stable internal reference gene, *Ubiquitin 1* (*UBQ1*; *AT3G52590*; Kobayashi et al., 2007, 2014). We have checked the invariant expression of *UBQ1* for all experimental condition in used lines in this study (Supplementary Figure S3). We included a control with no reverse transcriptase to assess genomic DNA contamination, and the amplification efficiency of all primers was confirmed. The primer sequences used to amplify the selected genes are shown in Supplementary Table S2.

### Expression Genome-Wide Association Study of *PGIP1*

The eGWAS analyses were carried out using TASSEL v3.0 software following a mixed linear model (MLM; Bradbury et al., 2007) using a total of 160,748 genome-wide single nucleotide polymorphism (SNP) information from public databases (Atwell et al., 2010; Cao et al., 2011; Horton et al., 2012),^2^ ^3^ which excluded SNPs of missing data or those with less than 5% minor allele frequency, as described earlier (Nakano et al., 2020b). The heritability (*h*^2^) was estimated by the following formula *h*^2^ = (the additive genetic variance)/(the additive genetic variance + the residual variance). The suggestive SNPs were determined by quantile–quantile (Q–Q) plot analysis (Zhang et al., 2018) using a free statistics software,^4^ and the genes closest to the SNPs (Table 1) were identified using the TAIR 10 database.^5^

**Table 1:** GWAS identified SNPs with directly linked to protein-coding genes that were associated with *PGIP1* expression levels in the shoots of 83 *Arabidopsis thaliana* accessions under Al stress.

Chr.	Physical position		GWAS *p*-value	Lower expression group		Higher expression group		Directly associated gene of detected SNP	SNP location in the directly associated gene	Functionally candidate^‡‡^	Al-responsive expression^††^	Short description	Allele frequency^†^	Mean of RFC^‡^	Allele frequency^†^	Mean of RFC^‡^
1	3,476,243	7.46 × 10^−5^	71/G	0.34	12/A	0.54	AT1G10540	Intron		1.04	NAT8 (nucleobase-ascorbate transporter 8)
1	22,938,272	1.88 × 10^−4^	79/G	0.37	4/A	0.39	AT1G62050	Exon		1.14	Ankyrin repeat family protein
1	23,795,163	1.39 × 10^−4^	74/C	0.33	9/G	0.68	**AT1G64105**	Exon	○	1.23^*^	NAC027 (NAC domain containing protein 027)
2	8,175,062	1.75 × 10^−4^	17/T	0.36	66/C	0.37	**AT2G18880**	−1,324	○	1.01	VEL2 (vernalization5/VIN3-like 2)
2	9,317,842	2.59 × 10^−4^	9/C	0.29	74/A	0.38	**AT2G21850**	Exon	○	0.97	Cysteine/Histidine-rich C1 domain family protein
2	9,354,086	9.32 × 10^−5^	10/A	0.23	73/C	0.38	**AT2G21950**	Exon	○	0.98	SKIP6 (SKP1 interacting partner 6)
3	8,902,459	2.93 × 10^−4^	13/T	0.27	70/A	0.39	**AT3G24480**	Exon	○	0.74	LRX4 (leucine-rich repeat extension 4)
5	4,969,631	3.16 × 10^−4^	35/T	0.27	48/A	0.44	**AT5G15300**	Exon		2.40^*^	Pentatricopeptide repeat (PPR) superfamily protein
5	9,229,573	1.45 × 10^−4^	13/C	0.30	70/A	0.38	AT5G26300	Intron		0.64	TRAF-like family protein
5	9,241,705	1.44 × 10^−4^	17/G	0.28	66/A	0.39	AT5G26330	Exon		0.92	Cupredoxin superfamily protein
5	14,889,845	2.74 × 10^−4^	62/G	0.35	21/T	0.46	AT5G37500	Exon		0.77	GORK (gated outwardly-rectifying K^+^ channel)
5	15,560,442	1.99 × 10^−4^	9/A	0.28	74/G	0.37	**AT5G38860**	Intron	○	1.15	BIM3 (BES1-interacting Myc-like protein 3)
5	15,574,085	2.08 × 10^−4^	77/G	0.35	6/A	0.59	**AT5G38900**	Exon	○	2.61^*^	Thioredoxin superfamily protein
5	17,460,312	6.27 × 10^−5^	62/C	0.35	21/T	0.48	**AT5G43460**	Intron	○	0.98	HR-like lesion-inducing protein-like protein
5	20,724,766	5.60 × 10^−5^	73/T	0.34	10/A	0.59	**AT5G50940**	Intron	○	1.05	RNA-binding KH domain-containing protein
5	23,783,404	2.34 × 10^−4^	66/A	0.33	17/C	0.51	**AT5G58900**	Exon	○	1.24^*^	Homeodomain-like transcriptional regulator (R-R-type MYB protein)
5	23,790,818	1.47 × 10^−4^	70/A	0.34	13/G	0.53	**AT5G58910**	Intron		1.45^*^	LAC16 (laccase 16)

† Number of accessions for each SNP allele.

‡ Mean value of relative fold change (RFC) in PGIP1 expression for accessions carrying the tolerant or sensitive SNP allele.

†† The fold change between Al treatment and control. Asterisk indicates showed greater than 1.2-fold change (*p* < 0.05). The expression data in the shoot obtained from our previous microarray data (Sawaki et al., 2016).

‡‡ The circle indicates gene that may be functionally related to regulation of gene expression based on their GO term and publications. The GO term is shown in Supplementary Figure S6.

The most focused SNPs with a value of *p* < 10^–3.5^ in the GWAS for expression levels of PGIP1 in the shoots of 83 A. thaliana accessions under 24-h Al treatment are presented. SNP locations within the directly associated gene are shown as exon or intron or upstream (denoted as a minus sign). Gene symbols and a short description of each gene from the literature and the TAIR/Araport11 database are indicated. Bold type indicates information on a priori candidate genes.

### Bioinformatics of Genes Associated With Significant SNP_S_

Gene ontology (GO) analysis was performed using an online tool available in the TAIR database.^6^ Gene polymorphisms were mined from the 1,001 Genomes database^7^ and POLYMORPH database.^8^ The genes upregulating *PGIP1* expression in T87-cultured cells of *Arabidopsis* were identified by the Regulatory-network Research (RnR) database (Sakurai et al., 2014).^9^ Co-expression network analysis was conducted on the eGWAS-detected and RnR database-listed genes using the ATTED-II database (Obayashi et al., 2018).^10^ Cis-elements and corresponding transcription factors (TFs) of the promoters were predicted using PlantPAN 3.0 (Chang et al., 2008).^11^

### *In planta* Complementation Assay of STOP1

The STOP1 complementation *Arabidopsis* transgenic plant was constructed as described by Ohyama et al. (2013). STOP1 genomic DNA containing the promoter (−2,848 from the first ATG) and downstream (+626 from the stop codon) regions was cloned into a binary vector (promoterless pBIG2113SF). This construct was introduced into *Agrobacterium tumefaciens* strain GV3101 and transformed into *STOP1-*KO plants by the floral dip method (Clough and Bent, 1998). A T3 homozygous line was used for the experiments.

### DNA-Protein Binding Assay

The binding of STOP1 to double-stranded, synthetic promoter fragments was studied using an amplified luminescent proximity homogeneous assay (AlphaScreen^™^, PerkinElmer, Waltham, MA, United States) and a 276 EnSpire Multimode Plate Reader (PerkinElmer) as described by Enomoto et al. (2019). Competition assays were performed with non-biotinylated probes (450 nM) according to the manufacturer’s instructions. The competitor probes, consisting of the promoter fragments −193 to −222 bp upstream from the *PGIP1* and − 2,694 to −2,723 bp upstream from the *AT5G38900* start codons, respectively, were designed around the STOP1-binding site according to the Plant Cistrome Database (Sadhukhan et al., 2019).^12^ The mutated probes were designed following the method of Tokizawa et al. (2015). The forward and reverse probe sequences are listed in Supplementary Table S3.

### Al Content in Pectin and Nuclear Magnetic Resonance Analysis

Pectin was extracted from 250 mg powdered *Arabidopsis* shoot tissue (500 seedlings were grown for 10 days in the hydroponics system mentioned above) in buffer containing 50 mM Tris–HCl (pH 7.2) and 50 mM cyclohexane-trans-1, 2-diamine tetra-acetate (CDTA; Bethke and Glazebrook, 2014). The extraction was continued for 15 min at 95°C with intermittent vortexing, and the sample was then centrifuged at 10,000 × *g* for 10 min. The supernatant containing pectin was analyzed for Al content using inductively coupled plasma mass spectrometry as described by Watanabe et al. (2015). For nuclear magnetic resonance (NMR) analysis, the supernatant containing pectin was lyophilized and dissolved in D_2_O. ^1^H-^13^C-Heteronuclear Single Quantum Coherence (HSQC) NMR was performed at 600.17 MHz on a JEOL ECA 600 NMR spectrometer (JEOL, Tokyo, Japan) equipped with a 5-mm FG/TH tunable probe, using the pulse sequence ‘hsqc_dec_club_pn’. NMR measurements were recorded at 70°C (Siedlecka et al., 2008). Sweep widths of 15 and 170 ppm were used to acquire the ^1^H and ^13^C spectra, respectively. For each NMR experiment, 88 scans were collected using a relaxation delay of 1.5 s.

## Results

### Genome-Wide Association Study to Detect Loci Associated With Expression of *Arabidopsis thaliana PGIP1* Under Al Stress

**Figure 1:**
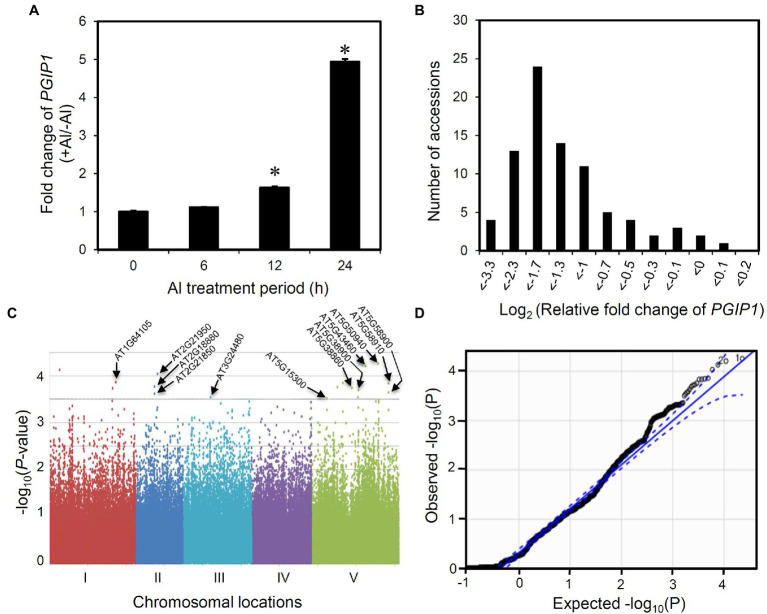
Expression GWAS for *POLYGALACTURONASE-INHIBITING PROTEIN 1* (*PGIP1*) expression in *Arabidopsis thaliana*. **(A)** Fold induction of *PGIP1* expression under Al stress at different time points of treatment are shown. The shoots were excised from 10-day-old approximately 100 seedlings of *Arabidopsis* (WT, Col-0) treated with either 0 or 25 μM Al for 0, 6, 12, and 24 h. Fold induction was calculated as the ratio of gene expression in growth media with Al to that without Al. An asterisk indicates significant difference from 0 h (Student’s *t*-test, *p <* 0.05). **(B)** Histogram showing the frequency distribution of *PGIP1* expression levels in the shoots of 83 *A. thaliana* accessions. 10 days pre-grown approximately 100 seedlings of each accession were treated with 25 μM Al for 24 h for quantification of gene expression. *UBQ1* was used as an internal control in the qRT-PCR experiment. The average data of three technical replicates are shown as relative fold change of *PGIP1* expression compared to Col-0. **(C)** GWAS of *PGIP1* expression levels in 83 *A. thaliana* accessions. The results are presented as a Manhattan plot of significance [−log_10_ (value of *p*)] versus chromosomal locations of SNPs. The horizontal dashed line indicates a value of *p* threshold according to the Q–Q plot of GWAS from **D**. Locations of the *a priori* candidate genes directly associated with the suggestive SNPs (*p* < 10^–3.5^; see also Table 1) based on the Q–Q plot of GWAS are shown by arrows. **(D)** Q–Q plot of GWAS, straight line represents expected null distribution of values of *p*; dots represent observed distribution of values of *p*; dotted line represents the 95% confidence intervals. Primers used for qRT-PCR are listed in Supplementary Table S2.

We analyzed the expression of *PGIP1* in the shoots of wild-type (WT) *Arabidopsis*, Columbia (Col-0) at different time points after root exposure to 25 μM Al. The expression of *PGIP1* was induced after 12 h and was markedly induced (five-fold on average) after 24 h of treatment (Figure 1A). It is also a condition of Al accumulation in the shoots (Supplementary Figure S4). Hence, in this study, we chose 24 h as the time point for evaluating *PGIP1* gene expression under Al stress to conduct the eGWAS of *PGIP1* in the shoots. Next, we analyzed the expression of *PGIP1* in 83 *Arabidopsis* accessions (Supplementary Figure S5; Supplementary Table S4) that had been treated with treated with 25 μM Al for 24 h and found that the expression range between log_2_ RFC −3.3 and log_2_ RFC 0.1 (RFC: relative fold change; compared to Col-0; Figure 1B; *h*^2^ = 88.3%). We performed a GWAS following MLM using the *PGIP1* expression data, but after Bonferroni correction for multiple testing, we could not detect any significant SNPs at the genome-wide significance level. This could be due to the dependence of the statistical power of GWAS on the population size and allele frequency. Although the Q–Q plot showed only a slight deviated plot, in this study we set a suggestive threshold based on this result (*p* < 10^–3.5^) and selected the top-ranked 17 SNPs as suggestive SNPs associated with *PGIP1* expression level variation (Figures 1C,D; Table 1). These potentially associated SNPs were selected for further analysis.

We first characterized 17 protein-coding genes carrying the 17 SNPs directly in their exons, introns, and promoters (Table 1) and then considered *a priori* candidate genes related to the regulation of *PGIP1* expression out of these 17 genes. Out of these, seven genes belonged to the GO term of “regulation of gene expression,” “DNA-binding transcription factor activity,” “intracellular signal transduction,” and “hormone-mediated signaling pathway” and may be functionally associated with *PGIP1* expression (Table 1; Supplementary Figure S6). Out of the *a priori* candidate genes, *AT2G21950* (*SKP1 interacting partner 6*: *SKIP6*; Farrás et al., 2001) and *AT3G24480* (*leucine-rich repeat extension 4*: *LRX4*; Zhao et al., 2018) are involved in hormonal signaling and cell-wall integrity, respectively. *AT5G38900* (*Thioredoxin superfamily protein*: *TRX SF*) is involved in regulation of gene expression *via* redox signaling (Sevilla et al., 2015). On the other hand, five among the 17 genes were induced more than 1.2-fold by Al in the shoot, according to our earlier transcriptome analysis, carried out under the same experimental conditions (Sawaki et al., 2016; Table 1). Therefore, we finally selected 12 genes with functions related to transcriptional regulation and Al-responsive expression, as *a priori* candidate genes associated with *PGIP1* expression, for subsequent analyses.

### Expression Level Polymorphisms and Amino Acid Polymorphisms Caused by Detected SNP of the Candidate Genes

**Figure 2:**
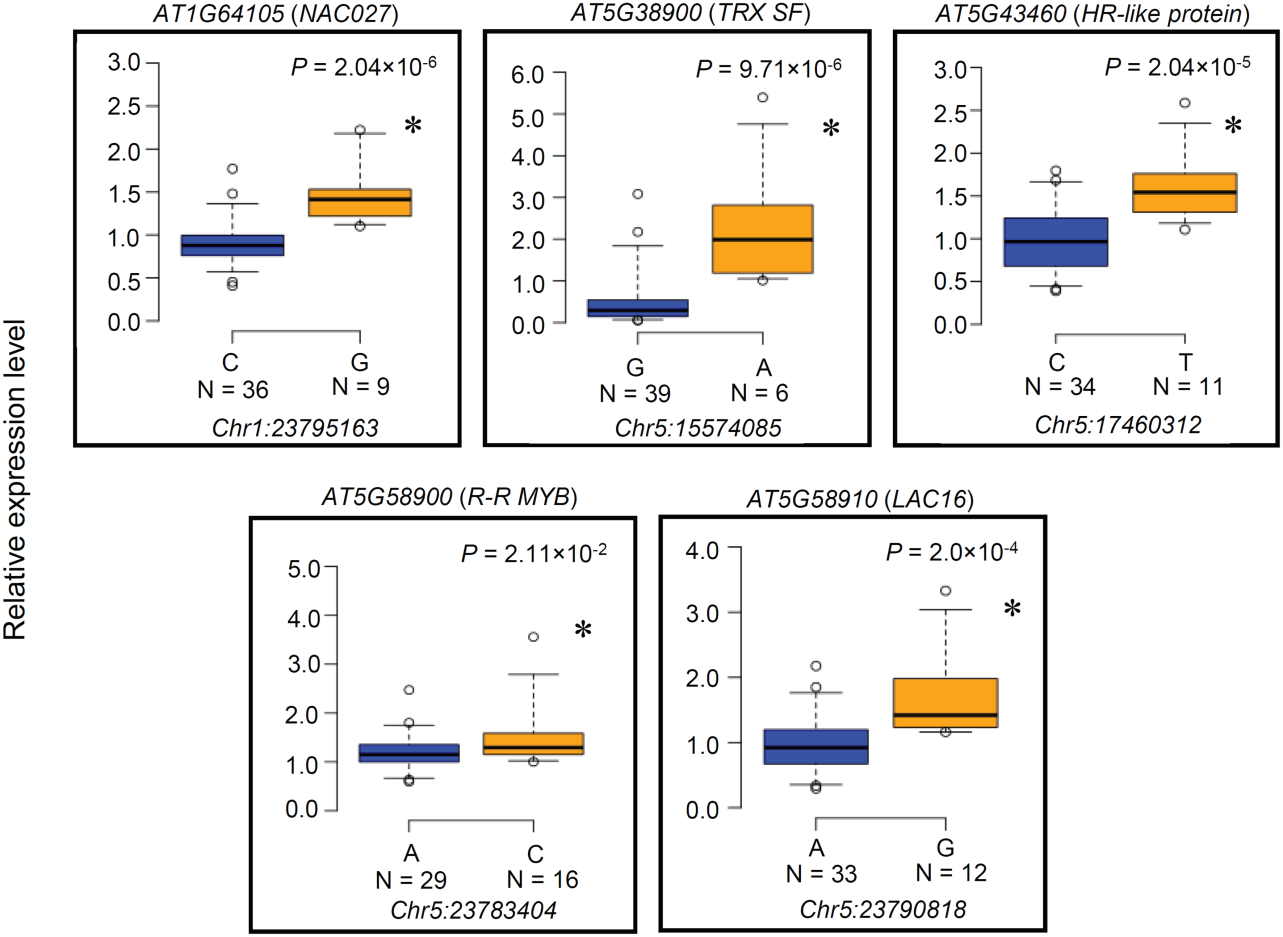
Expression level polymorphisms of the genes identified by expression genome-wide association study (eGWAS). The expression level variations in *a priori* candidate genes directly linked to SNPs (Table 1) that showed significant segregation between SNP alleles are presented as box plots. Genes whose expression levels were not significantly different between accessions are shown in Supplementary Figure S7. Expression of these genes was monitored by qRT-PCR in 45 randomly chosen accessions among the 83 accessions used in eGWAS. Accessions are grouped according to SNP alleles at a particular physical chromosome position mentioned below each box plot. Number of accessions of each SNP allele is shown under the each SNP. *UBQ1* was used as an internal control. Expression level of each accession is relative to Col-0. An asterisk indicates a significant difference between the average values of group (*p* < 0.05, Student’s *t-*test).

We examined the 12 candidate genes for expression level polymorphisms (ELPs) and amino acid polymorphisms associated with the *PGIP1* expression level. The expression levels of these genes were compared between representative accession groups that carried different detected SNP alleles (Figure 2, Supplementary Figure S7). Five genes, *AT1G64105* (*NAC027*), *AT5G38900* (*TRX SF*) *AT5G43460* (*HR-like lesion-inducing protein-like protein*: *HR-like protein*), *AT5G58900* (*Homeodomain-like transcriptional regulator*: *R-R MYB*), and *AT5G58910* (*laccase 16*: *LAC16*) exhibited significant differences in the level of expression between accessions carrying different SNP alleles (Figure 2). When comparing the different alleles, the minor allele group with elevated *PGIP1* expression showed higher expression levels of each gene than the major allele group with low *PGIP1* expression (Table 1; Figure 2). In the case of genes with SNP alleles located directly in exons, we examined the amino acid polymorphisms caused by the detected SNPs using reliable DNA sequences from the 1,001 genome database. The SNPs detected in the exons of five genes caused amino acid polymorphisms, *viz*. Ser29Cys in *NAC027*, Ala187Ser in *SKIP6*, Asn60Lys in *LRX4*, Phe134Ile in *AT5G15300* (*pentatricopeptide repeat superfamily protein*) and His246Gln in *R-R MYB*. Among them, *NAC027* and *R-R MYB* showed both ELP and amino acid polymorphism. These polymorphisms might affect the expression level variation of *PGIP1* as *cis*-factors. In this way, eight genes were selected as the first group of possible candidate genes, which were further studied using reverse genetics.

### Reverse Genetic Characterization of the Candidate Genes

**Figure 3:**
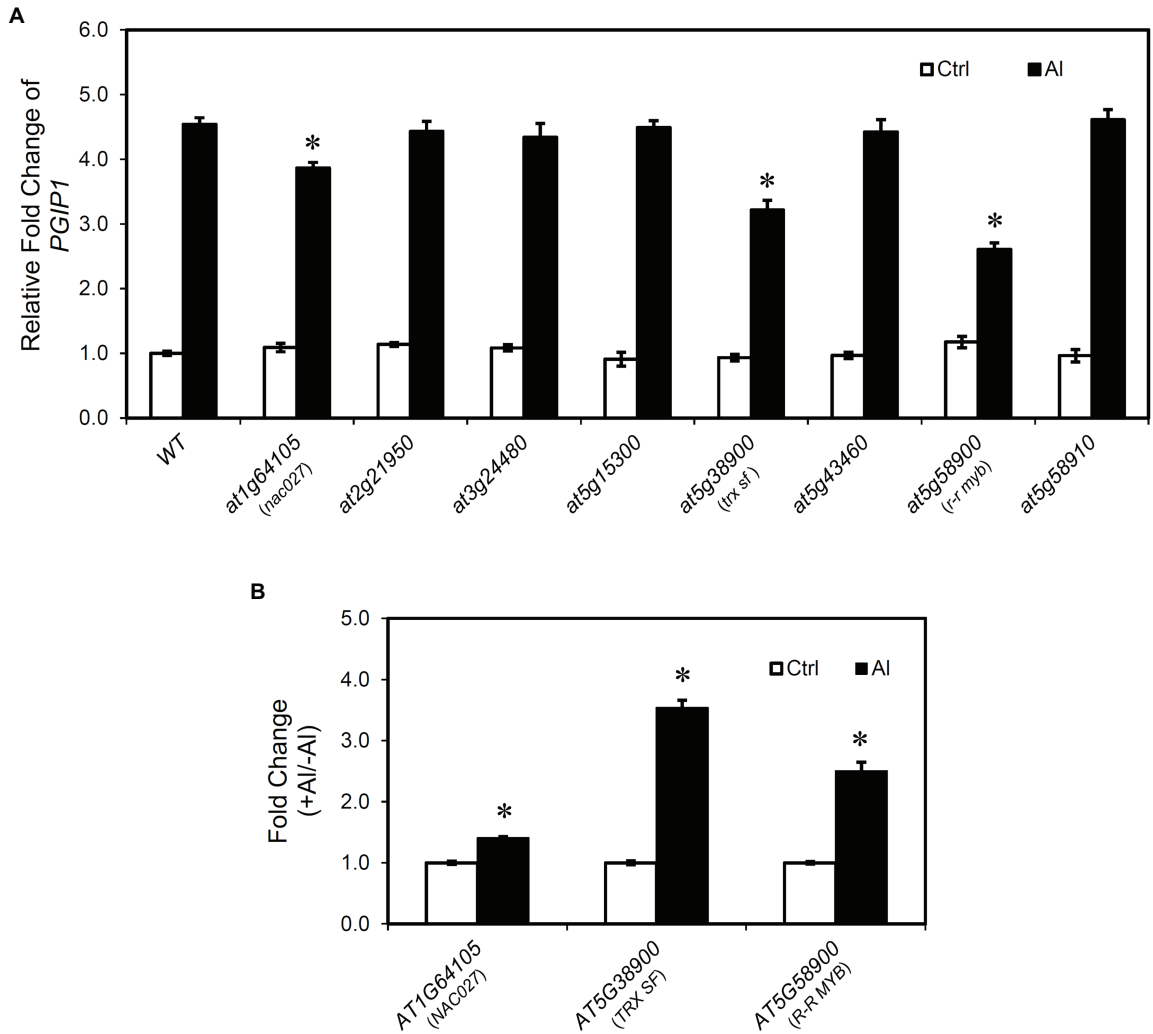
*PGIP1* expression in the mutants of genes identified by eGWAS. **(A)** Relative expression levels of *PGIP1* in the shoots of WT (WT, Col-0) and T-DNA insertion mutants of eight final candidate genes having expression level polymorphisms (Figure 2) and/or amino acid polymorphisms (see main text). Approximately 100 seedlings of the WT and independent homozygous T-DNA insertion mutants were grown for 10 days and treated with 0 (-Al) or 25 μM AlCl_3_ (+Al) for 24 h. *PGIP1* expression was measured by qRT-PCR. Expression levels are expressed as relative fold changes compared to WT (-Al); significant reductions in relative fold change of *PGIP1* from the Al-treated WT sample are indicated by asterisks (Student’s *t-*test, *p <* 0.05). **(B)** Fold changes (+Al/-Al) of *AT1G64105* (*NAC027*), *AT5G38900* (*TRX SF*), and *AT5G58900* (*R-R MYB*) at 24 h of Al treatment, measured by qRT-PCR, are shown; significant fold increases from the control (-Al) samples are indicated by asterisks, ^*^(Student’s *t*-test, *p* < 0.05). Average data of three biological replicates are presented with standard errors. *UBQ1* was used as an internal control. Primers used for qRT-PCR are listed in Supplementary Table S2.

To examine the effect of the candidate genes on *PGIP1* expression, its expression level was quantified in the T-DNA insertion knockout (KO) or knockdown mutants (KD; Supplementary Figure S8) of the eight candidate genes. *PGIP1* expression was significantly lower in the three T-DNA insertion mutants of *nac027*, *trx sf* and *r-r myb* than in the WT under Al treatment (Figure 3A). In particular, the reduction of *PGIP1* expression in the *r-r myb* was as high as approximately 40%. In contrast, other mutants (*at2g21950*, *at3g24480*, *at5g15300*, *at5g43460*, *at5g58910*) showed no significant difference of *PGIP1* expression compared to the WT under Al treatment (Figure 3A). From this analysis, we found that *NAC027*, *TRX SF* and *R-R MYB* are involved in the regulation of *PGIP1* expression.

We also analyzed the patterns of expression of *NAC027*, *TRX SF*, and *R-R MYB* under Al stress in the shoots of WT *A. thaliana* after 24 h of treatment. We observed a significant induction of these genes relative to the control (Figure 3B). The Al-induced responses of *NAC027* were weak, but their gene expression showed Al responses similar to *PGIP1* expression. These results suggest that *NAC027*, *TRX SF*, and *R-R MYB* are related to the regulation of Al-induced *PGIP1* expression. In addition, Al-responsive *PGIP1* and its regulatory system were involved in Al tolerance. In acidic soils containing higher exchangeable Al, *pgip1*, like the Al-hypersensitive *stop1*, was much more inhibited in growth than the WT (Supplementary Figure S9). On the other hand, the growth was recovered in the neutralized soil (Supplementary Figure S9). Similarly, the growth of *trx sf* and *r-r myb* was inhibited than the WT in acidic soil, although the growth of *nac027* was not severely inhibited (Supplementary Figure S9). This may be consistent with the lower degree of repression of *PGIP1* in *nac027* and the weaker induction of Al on the expression level of *NAC027* compared to the other two genes (Figure 3).

### Relationship Between *STOP1* and Genes Regulating *PGIP1* Identified by eGWAS

**Figure 4:**
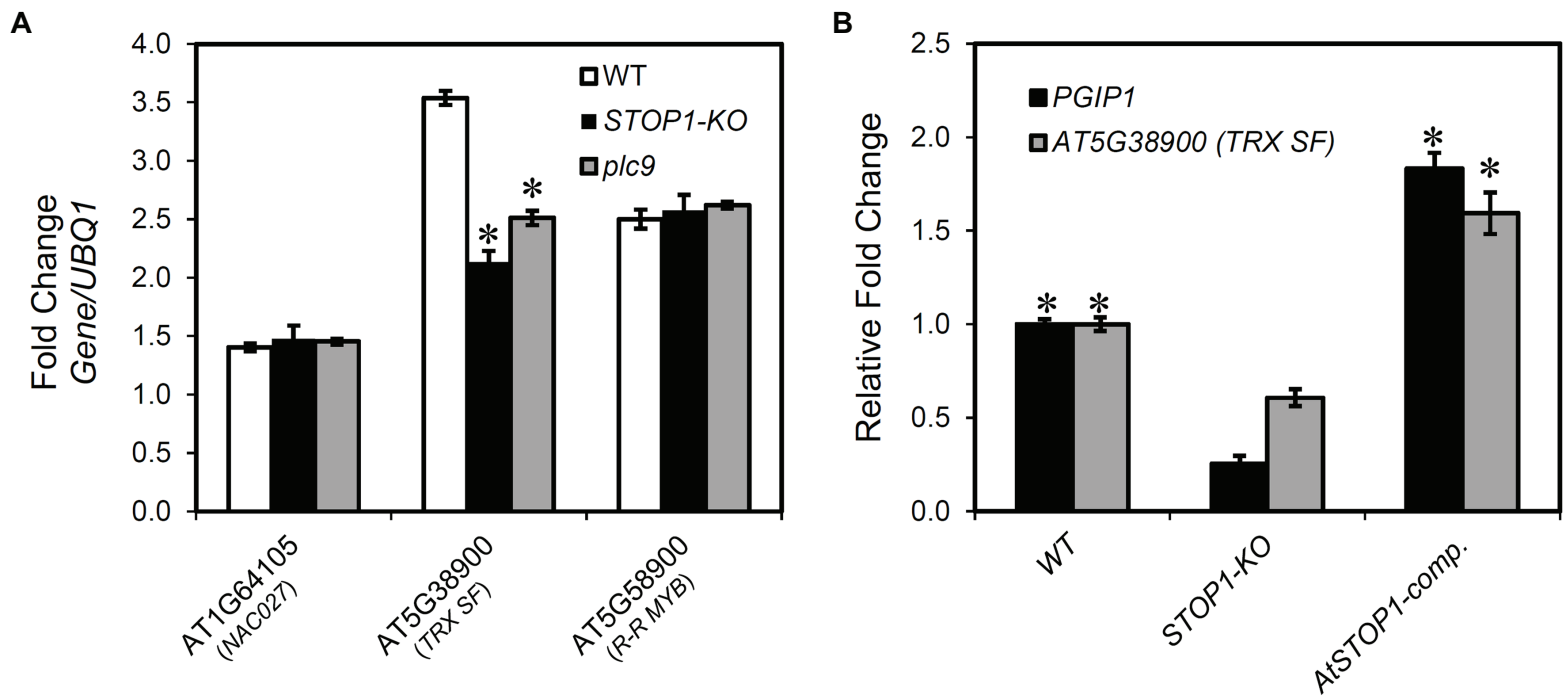
Relationship of SENSITIVE TO PROTON RHIZOTOXICITY 1 (*STOP1*) to the genes identified by eGWAS for *PGIP1* expression. **(A)** Transcriptional regulation of the genes identified in eGWAS (*NAC027*, *TRX SF*, and *R-R MYB*) by STOP1 and PLC9 was studied. Seedlings of WT (WT, Col-0) and knockout (KO) lines were grown for 10 days in MGRL solution and treated with 25 μM AlCl_3_ for 24 h. The expression levels of *NAC027*, *TRX SF*, and *R-R MYB* were measured in the shoots of WT, *STOP1*-KO, and *plc9* by qRT-PCR. An asterisk indicates a significant difference from WT (Student’s *t-*test, *p <* 0.05). **(B)** Recovery of the transcription of suppressed *PGIP1* and *TRX SF* in *STOP1-KO* was analyzed in complemented line (*AtSTOP1-comp*.) after exposure to Al (25 μM AlCl_3_ for 24 h). Gene expression levels are relative to WT and fold changes from the control (-Al) are shown. An asterisk indicates a significant difference from *STOP1-*KO (Student’s *t-*test, *p <* 0.05). *UBQ1* was used as an internal control. Average values of three biological replicates are presented with standard errors.

We investigated the relationship between STOP1 regulation and the eGWAS-detected genes because it has been reported that STOP1 regulates *PGIP1* expression (Sawaki et al., 2009). There was no difference in expression levels of *STOP1* in the *nac027*, *trx sf*, and *r-r myb* (Supplementary Figure S10A). In contrast, we found a significant reduction in the gene expression level of *TRX SF* in the *STOP1*-KO, whereas the other two genes showed similar expression levels compared to WT (Figure 4A). In addition, in the STOP1-complemented line, the expression of *TRX SF* was fully recovered similar to Col-0 (Figure 4B). These results suggest that *TRX SF* is regulated by the STOP1 transcription factor, while none of the three genes affect the expression level of *STOP1*.

In our previous eGWAS of *ALS3*, we found the involvement of *phosphoinositide* (*PI*)*-dependent phospholipase C9* (*PLC9*) signaling upstream of the STOP1 regulation system, which regulates the expression of *ALS3* and *PGIP1* in the shoots of *A. thaliana* (Sadhukhan et al., 2020). Therefore, we examined the expression of *TRX SF*, *NAC027*, and *R-R MYB* in the *plc9*. We found that the expression of *TRX SF* was significantly suppressed in *plc9* (Figure 4A), similar to the downregulation of *PGIP1* (Supplementary Figure S10B). These findings suggest that *TRX SF* is regulated by a PI signaling-mediated STOP1-dependent pathway. In contrast, we found that the expression of *ALS3* remained unchanged in the *trx sf*, *nac027*, and *r-r myb* mutants (Supplementary Figure S10A). These results suggest that *TRX SF* differentially regulates transcription of *PGIP1* and *ALS3*, which are co-regulated by STOP1 in *Arabidopsis* shoots.

### *In vitro* Binding Analysis of STOP1 to *TRX SF* and *PGIP1* Promoter Regions

**Figure 5:**
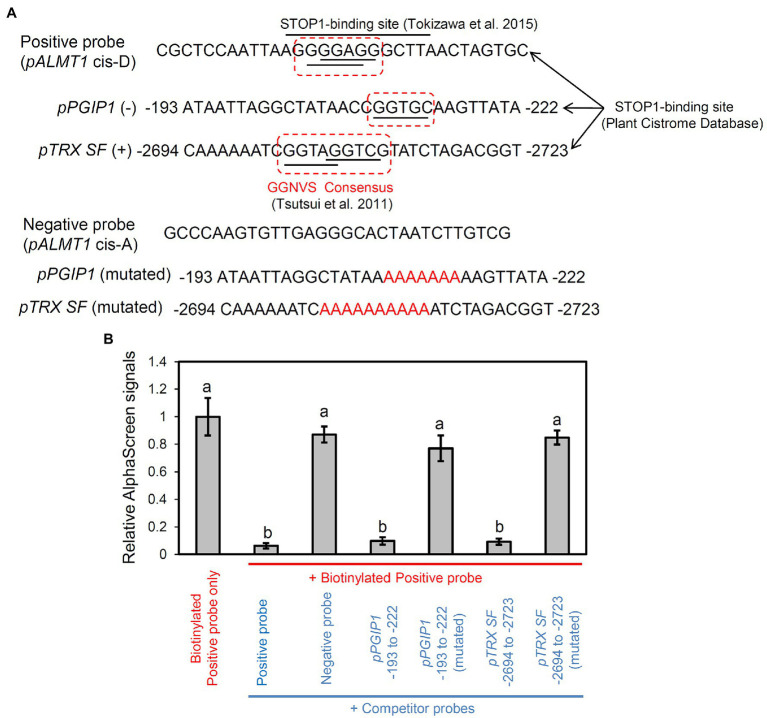
Binding assay of STOP1 protein with *PGIP1* and *TRX SF* promoters. Results of competitive binding assay of synthetic double-stranded promoter fragment of *PGIP1* (−193 to −222 bp) and *TRX SF* (−2,694 to −2,723) to *in vitro* translated STOP1 protein using the PerkinElmer Amplified Luminescent Proximity Homogeneous Assay (AlphaScreen^™^) are shown. **(A)** Probe sequences used in AlphaScreen are shown. The STOP1-binding sites of the *PGIP1* and *TRX SF* promoters were chosen from the Plant Cistrome Database (http://neomorph.salk.edu/dap_web/pages/index.php). The dotted boxes indicate the GGNVS sequences that represent the STOP1-ortholog ART1 binding minimum consensus. Known binding (*cis*-D) and non-binding (*cis*-A) sites of STOP1 on the *AtALMT1* promoter (Tokizawa et al., 2015) were used as positive and negative control probes, respectively. The mutated *PGIP1* and *TRX SF* probes were designed by replacing the GGNVS consensus sequence with stretches of A/T (shown in red font). **(B)** Competitive binding assay where 450 nM competitor probes (shown in blue font) compete with 50 nM biotinylated positive probe (shown in red font) for binding with the STOP1 protein. The emitted light signal intensities, relative to those of the biotinylated probe without any competitor, are shown in the graph. Average values ± SD (n = 3) are presented. Lower emitted signal intensity signifies binding of the STOP1 protein to the respective competitor probe. Different letters indicate significant differences of emission intensity (Tukey’s test, *p* < 0.05).

Next, we performed a promoter binding analysis to reveal whether STOP1 directly regulates *TRX SF* and *PGIP1* in the STOP1-dependent pathway. The STOP1-binding positions were predicted by searching enriched sequences (octamer units) in the stress-inducible promoters.^13^ They were identical to the binding sites provided by the Plant Cistrome Database. Therefore, we searched for putative STOP1-binding sites in the promoters of *PGIP1* and *TRX SF* using the Plant Cistrome Database. Based on DNA affinity purification sequencing (DAP-seq), the database identified the “GGNVS” consensus sequence in the *PGIP1* and *TRX SF* promoters for binding STOP1-like proteins (Figure 5A), as previously identified in rice by Tsutsui et al. (2011). The binding capacity of STOP1 to the sequences available from the Plant Cistrome Database was validated by an *in vitro* competitive binding assay using the AlphaScreen^™^ system (Figure 5B). A 30-bp synthetic double-stranded DNA probe, designed around the binding site in the *PGIP1* (−193 to −222 bp) and *TRX SF* (−2,694 to −2,723) promoters, could compete for STOP1 protein binding with a known STOP1-binding site in the *AtALMT1* promoter (Tokizawa et al., 2015). On the other hand, replacing the “GGNVS” consensus sequences within the *PGIP1* and *TRX SF* probe with A/T stretches abolished STOP1 binding (Figure 5B). These results indicate that STOP1 binds directly to the *PGIP1* and *TRX SF* promoters.

### Al-Inducible NO Signaling Effects Expression of Genes Regulating *PGIP1*

**Figure 6:**
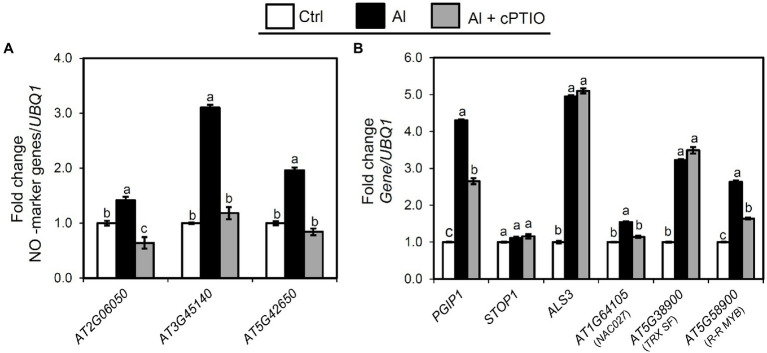
Relationship between Al stress and endogenous NO signaling. Ten-day-old seedlings were exposed to 25 μM Al solution containing 0 or 50 μM cPTIO (NO scavenger) for 24 h. **(A)** Expression analysis of NO marker genes in shoots after exposure to Al. **(B)** Effect of cPTIO on expression of the genes identified in eGWAS (*NAC27*, *TRX SF*, and *R-R MYB*), *PGIP1*, *STOP1*, and *ALS3* in shoots after 24 h Al exposure. *UBQ1* was used as an internal control. Average values of three biological replicates are presented with standard errors. Fold change was calculated from control (−Al). Primers used for qRT-PCR are listed in Supplementary Table S2. Different letters indicate a significant difference at *p* < 0.05 (Tukey test).

NO generation is positively correlated with cell wall pectin demethylation and alteration of cell wall metabolism under Al stress (Zhou et al., 2012; Sun et al., 2016). To establish whether Al accumulation induces NO signaling, we examined NO-inducible marker gene (*AT2G06050*, *AT3G45140*, and *AT5G42650*; Huang et al., 2004) expression in shoots after exposing the roots to Al or Al plus cPTIO (NO scavenger; Shi et al., 2015) for 24 h. We found that the expression of the NO marker genes was substantially induced in the Al-treated samples, whereas their expression was suppressed in the samples treated with Al plus cPTIO (Figure 6A). This suggests that Al induces NO signaling in the shoots. Next, we examined whether *PGIP1* and *ALS3*, which are regulated by STOP1, function downstream of NO signaling by quantifying their expression levels in the shoots after exposing the roots to Al or Al plus cPTIO for 24 h. The transcript levels of *PGIP1* were suppressed significantly in the Al plus cPTIO samples, whereas *ALS3* expression was unchanged (Figure 6B). STOP1 expression was neither Al-induced nor affected by cPTIO.

We also assessed whether the eGWAS-identified genes that regulate *PGIP1* expression function downstream of NO signaling by quantifying their expression levels in the shoots after exposing the roots to Al or Al plus cPTIO for 24 h. We found that only *NAC027* expression and *R-R MYB* expression were significantly suppressed in the Al plus cPTIO samples, while *TRX SF* remained unchanged (Figure 6B). These results clearly indicate that Al-inducible *PGIP1* expression is regulated by the NO signaling pathway through *NAC027* and *R-R MYB*, and this is not regulated by STOP1 (Figure 4A).

### Transcriptional Regulation of *R-R MYB* and *NAC027* Included in NO Signaling

**Figure 7:**
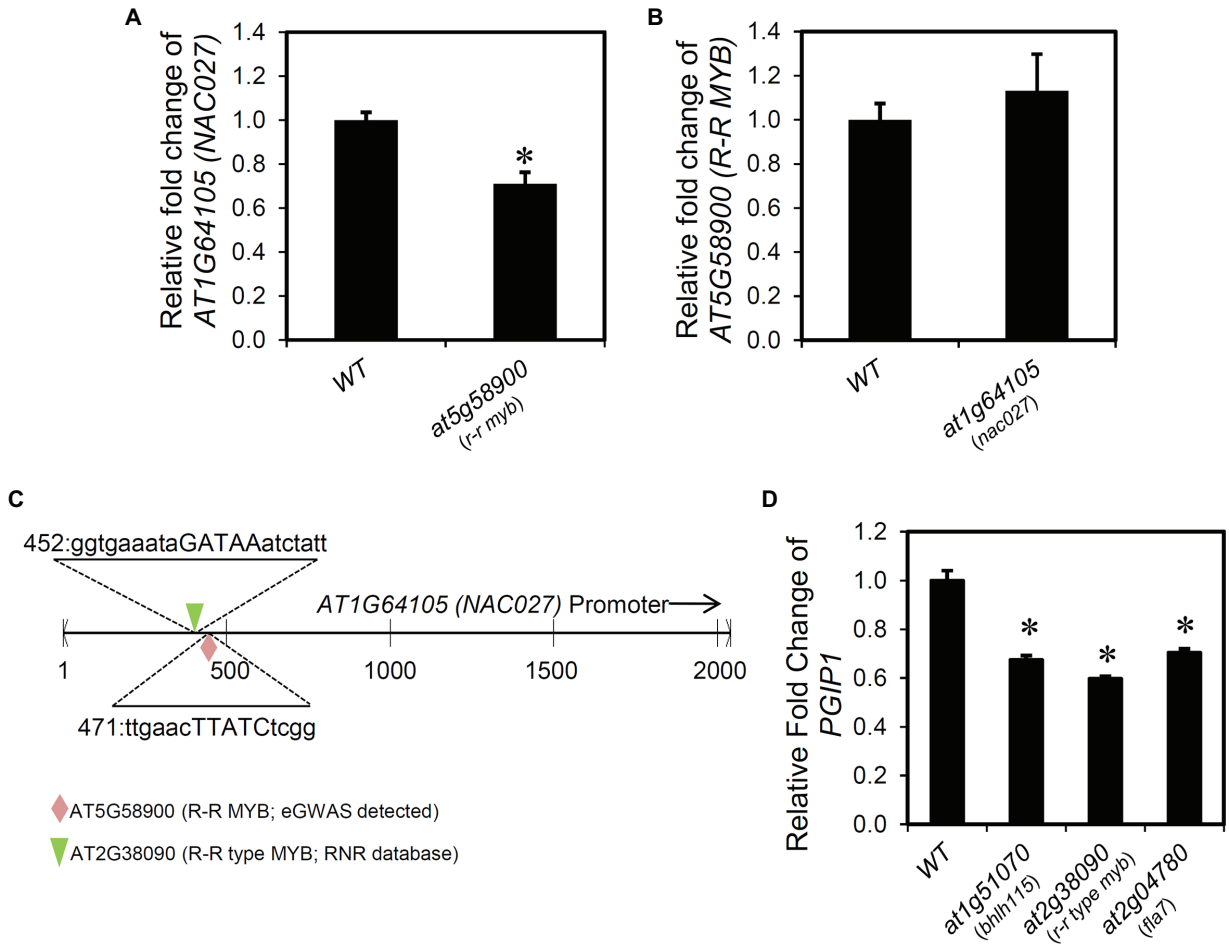
Transcriptional regulation of the genes regulating *PGIP1*. **(A)** Relative expression levels of *NAC027* in the shoots of WT and *r-r myb*. **(B)** Relative expression levels of *R–R MYB* in the shoots of WT and *nac027*. **(C)** AT5G58900 (*R-R MYB*; eGWAS detected) and AT2G38090 (*R-R type MYB*; RNR database detected) binding position to the *NAC027* (*AT1G64105*) promoter region retrieved from PlantPAN 3.0 and the corresponding sequence. **(D)** Relative expression levels of *PGIP1* in the shoots of WT and T-DNA insertion mutants of genes (*at1g51070*, *at2g38090*, and *at2g04780*) reported in RNR database that regulate *PGIP1* expression. Approximately 100 seedlings of WT and independent homozygous T-DNA insertion mutants were grown for 10 days and treated with 25 μM AlCl_3_ for 24 h. For *NAC027*, *R-R MYB*, and *PGIP1*, expression was measured by qRT-PCR. Expression levels are expressed as relative fold changes compared to WT. Error bars indicate standard errors across three biological replicates. *UBQ1* was used as an internal control. Significant reductions compared to the Al-treated WT sample are indicated by asterisks (Student’s *t-*test *p <* 0.05). Primers used for qRT-PCR are listed in Supplementary Table S2.

The expression of *NAC027* and *R-R MYB* was measured in each KO line. A significant reduction in the expression of *NAC027* was observed in the *r-r myb* compared with the WT expression (Figure 7A). In contrast, the expression of *R-R MYB* in the *nac027* showed no difference compared with wild type (Figure 7B). In addition, the PlantPAN3.0 database (provides TF binding sites in genome-wide promoters based on DAP-seq analysis of various transcription factors) identified that R-R MYB directly binds to the promoter of *NAC027* (Figure 7C).

Interestingly, we found regulators of *PGIP1* using the RnR database that is different from the eGWAS-identified factors. The RnR database indicated that overexpression of *AT1G51070* [*BASIC HELIX-LOOP-HELIX 115* (*BHLH115*)], *AT2G38090* (duplicated homeodomain-like superfamily protein), and *AT2G04780* [*FASCICLIN-LIKE ARABINOGALACTAN 7* (*FLA7*)] upregulated *PGIP1* expression, which was at the approximately 99th percentile of expression regulation and about 1.3–2.0-fold expression compared with the control. In fact, we confirmed the downregulation of *PGIP1* expression in the KO or KD mutants of these three genes under Al stress (Figure 7D). Among them, *AT2G38090* is a member of the R-R-type MYB family (Yanhui et al., 2006) and is a close homolog of R-R MYB that we found in the eGWAS. This TF also binds to the *NAC027* promoter as assessed by a PlantPAN3.0 database search, similar to R-R MYB (Figure 7C). Co-expression gene network analysis using the six genes (*TRX SF*, *NAC027*, *R-R MYB*, *BHLH115*, *AT2G38090*, and *FLA7*), whose *PGIP1* expression levels were decreased in these KO or KD lines, revealed that *R-R MYB* was co-expressed with *AT2G38090* (*R-R type MYB*) and *FLA7* (Supplementary Figure S11). These results suggest that these genes are involved in the direct/indirect regulation of *PGIP1* expression.

## Discussion

**Figure 8:**
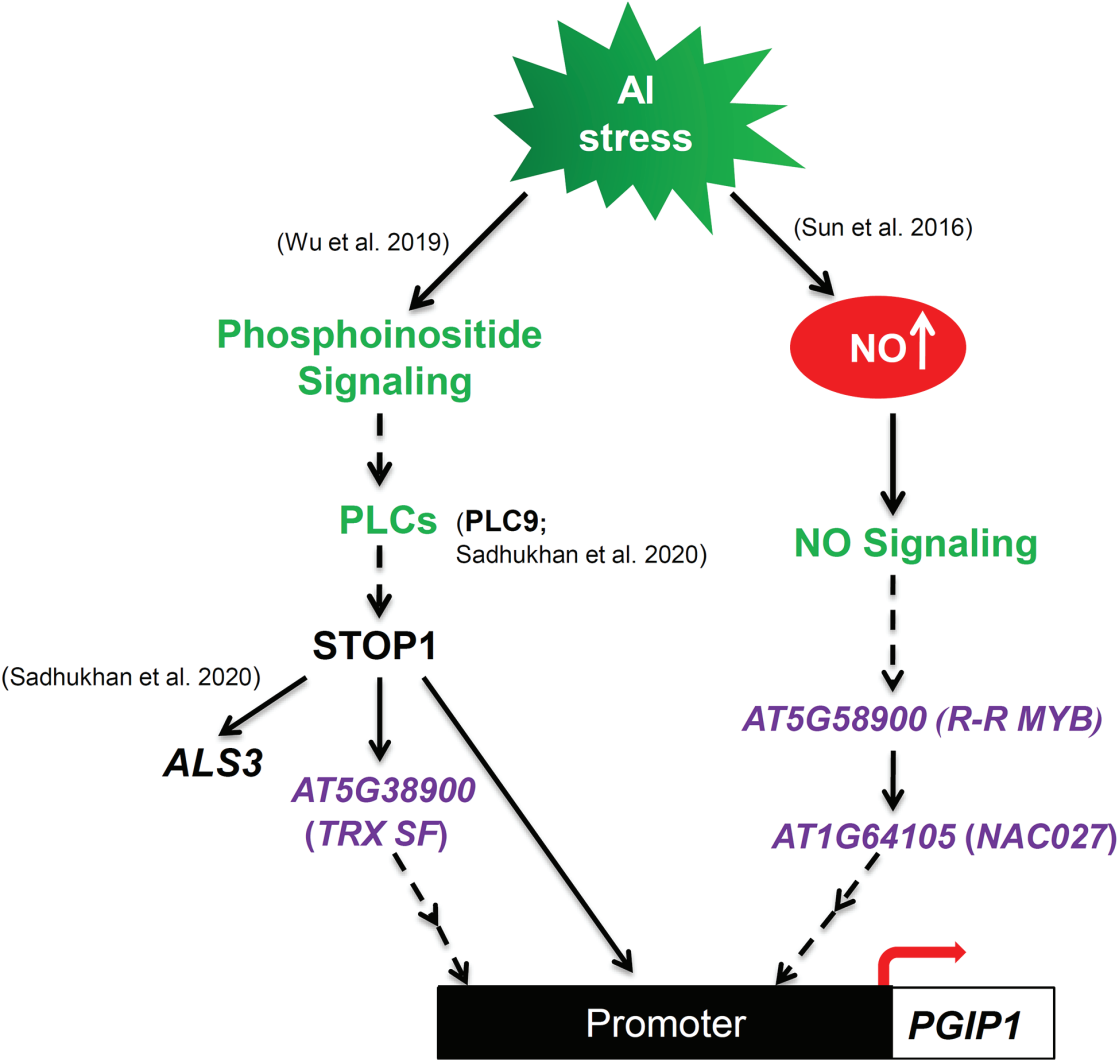
Schematic illustration of proposed model illustrating that Al-induced, STOP1-independent NO-signaling pathway and Al-activated STOP1 signaling cascades regulate *PGIP1* expression under Al stress. *TRX SF*, *R-R MYB*, and *NAC027* were identified by eGWAS for *PGIP1* expression levels under Al stress in the shoots of *A. thaliana*. The colors green and purple represent the previously known Al signaling pathways and newly discovered genes in this study, respectively.

The transcription of Al-tolerance genes is regulated by a complex mechanism (Delhaize et al., 2012) and involves STOP1 and cross talk with other mechanisms related to stress responses (Daspute et al., 2017). In this study, *TRX SF*, *R-R MYB*, and *NAC027* involved in the regulatory mechanisms of Al-inducible *PGIP1* expression were identified through eGWAS of *PGIP1* expression levels under Al stress (Figure 3; Table 1). The STOP1-TRX SF pathway regulate *PGIP1* expression through the PI signaling pathway *via* PLC9, while R-R MYB and NAC027 regulate *PGIP1* expression through a STOP1-independent NO-signaling pathway (Figures 4, 6, 8). In contrast, the regulation of *ALS3* expression *via* STOP1 in the shoots was independent of these pathways, including *TRX SF*, *R-R MYB*, and *NAC027* (Figures 6, 8, Supplementary Figure S10). Taken together, the eGWAS of *PGIP1* identified a portion of the complex Al signaling pathways in *Arabidopsis* shoots.

In the current study, we found plausible causative genes involved in the regulation of *PGIP1* expression through the candidate gene-based GWAS (Table 1). Many of these genes showed genomic polymorphisms, but further reverse genetic studies clearly revealed that three genes, *TRX SF*, *R-R MYB*, and *NAC027*, were involved in the regulation of *PGIP1* expression. The polymorphism responsible for the variation could not be determined in this study. However, using reliable DNA sequences from the 1,001 Genomes Project, we searched for polymorphisms at the three genes in several high- and low-expression accessions. In this process, we observed a haplotype containing polymorphisms in the intron sequences of *TRX*, associated with its expression levels. The only amino acid polymorphism caused by the detected SNPs was observed in *R-R MYB*. Another haplotype including a promoter deletion related to ELP was observed in *NAC027* (Supplementary Figure S12). Promoter polymorphisms have been found to significantly impact gene expression level variation (Sadhukhan et al., 2017; Meloa et al., 2019; Nakano et al., 2020b; Wang et al., 2021). Functional analysis of the promoter sequence polymorphisms of *NAC027* will shed light on their potential role in the differential regulation of gene expression.

A key regulatory transcription factor, STOP1, regulates the expression of various genes involved in Al tolerance along with *PGIP1* (Sawaki et al., 2009; Wu et al., 2019), in which some genes are directly regulated. Our previous studies found that STOP1 directly regulates transcription of several downstream genes by binding to their promoters under not only Al but also other stress conditions: STOP1 binds to the promoter of Al-inducible *AtALMT1* (Tokizawa et al., 2015) and *AtMATE* (Nakano et al., 2020b), low-oxygen-inducible *HsfA2* (Enomoto et al., 2019), and NaCl-inducible *CPK23* (Sadhukhan et al., 2019). In the present study, promoter analyses (i.e., Cistrome database and *in vitro* promoter assays; Figure 5) identified a functional STOP1-binding site in the promoters of *PGIP1* and *TRX SF*. This indicates that STOP1 directly activates the Al-inducible expression of *PGIP1* and *TRX SF*. The promoter regions of each gene commonly possessed the minimum consensus sequence of ART1/STOP1 [GGN(T/g/a/C)V(C/A/g)S(C/G); Tsutsui et al., 2011; Tokizawa et al., 2015], although the surrounding sequences of the consensus were different.

Similarly, we found that STOP1 may directly regulate *TRX SF* expression (Figures 4, 5), while TRX SF affected the *PGIP1* expression but not *STOP1* expression (Figure 3, Supplementary Figure S10), whose regulation contributed to Al tolerance (Supplementary Figure S9). One possible function of TRX SF is to control regulatory proteins by oxidative protein modification, which is a common mechanism of thioredoxin superfamily proteins (Lemaire and Miginiac-Maslow, 2004; Schmidtmann et al., 2014; Mata-Perez and Spoel, 2019). The TRX family regulates gene expression through redox activation of receptors and transcription factors under salicylic acid (SA) and brassinosteroid signaling (Ding et al., 2018; Tian et al., 2018). We previously revealed that *TRX1* contributed to Al tolerance using GWAS for Al tolerance in *Arabidopsis* (Nakano et al., 2020a). These results suggest that TRX-mediated redox signaling is involved in gene regulation related to Al tolerance. However, *ALS3* was not involved in the signaling (Supplementary Figure S10), despite the fact that *ALS3* expression in shoots is regulated by STOP1 under Al stress, similar to that of *PGIP1* (Sawaki et al., 2016).

We found that both NAC027 and R-R MYB (transcription factors respond to various biotic and abiotic stresses, Ascencio-Ibanez et al., 2008; Soitamo et al., 2008; Fang et al., 2018) responded to Al stress and were involved in STOP1-independent regulation of *PGIP1*, where they function together in the NO signaling pathway *via* R-R MYB binding to the *NAC027* promoter (Figures 3, 4, 6, 7). However, the contribution of *NAC027* to regulation and Al tolerance does not seem to be largely comparable to that of *R-R MYB* (Figure 3, Supplementary Figure S9), suggesting that *R-R MYB*, upstream of *NAC027*, also regulates other genes. NO signaling has been reported to be a second messenger of Al-inducible expression of several Al-tolerance genes (He et al., 2012). Additionally, *PECTIN METHYLESTERASE 3*, which is induced and activated by Al-dependent NO signaling (Sun et al., 2016; Ye et al., 2018), was detected as a co-expressed gene of *R-R MYB* and the close homolog of *MYB* regulating *PGIP1* (Figure 7D, Supplementary Figure S11). These results suggest that the co-expressed module involved in NO signaling is related to the Al stress response. In contrast, STOP1 regulated *TRX SF* along with *ALS3* (Figure 6), which was independent of NO signaling not included in the co-expression network.

Furthermore, several genes involved in plant cell wall biogenesis, *UDP-GLUCOSE DEHYDROGENASE 4*, *COTTON GOLGI-RELATED 3*, and *FLA7*, were included in the co-expression network. The cell wall plays important roles not only in the regulation of plant growth and development, but also in the perception and expression of Al toxicity (Tabuchi and Matsumoto, 2001; Eticha et al., 2005; Yang et al., 2011; Kobayashi et al., 2013; Zhu et al., 2014; Kochian et al., 2015; Sun et al., 2016). The PGIP1 was shown to be included in Al tolerance (Supplementary Figure S9), but the details of its role in Al tolerance have not been studied yet; one possibility is that it can protect the binding of Al to negatively charged ligands [e.g., polygalacturonic acid (PGA)] induced by demethylation of cell wall pectin, which is enhanced by Al (Supplementary Figures S4, S13: the degree of pectin methylesterification in Al-treated seedlings decreased to 55% of that without Al treatment). It has been reported that PGIP1 can bind to the PGA region and support the formation of a normal pectin network under biotic stress conditions (Spadoni et al., 2006). A similar alleviation was observed under proton-toxic conditions in the *stop1*, which showed very low expression of *PGIP1* (Kobayashi et al., 2014). Under Al stress conditions, PGIP1 binding to the PGA region might reduce the formation of abnormal pectin networks, which might be caused by unusual Al binding to the PGA region. Further characterization of these events would be useful for identifying the role of PGIP1 in Al tolerance.

## Conclusion

Through a candidate gene-based GWAS of *PGIP1* expression, we successfully identified complex signaling of *PGIP1* in response to Al stress in *A. thaliana*. Furthermore, we propose a model to illustrate that *PGIP1* expression is regulated by a STOP1-dependent Al-induced phosphoinositide (PI) signaling through *AT5G38900* (*TRX superfamily protein*) and STOP1-independent Al-induced endogenous NO signaling through *AT1G64105* (*NAC027 transcription factor*) and *AT5G58900* (*R-R type MYB transcription factor*; Figure 8). In addition, our study demonstrates the utility of an eGWAS in understanding the genetic regulation of Al signaling by exploiting the natural variation in the expression levels of key Al-responsive genes. Although a limited number of accessions were used in the current study, a future eGWAS using denser SNP information and a larger accession set, available in recent years (Togninalli et al., 2018), will open up new avenues for better understanding of Al stress signaling in plants.

## Data Availability Statement

The datasets presented in this study can be found in online repositories. The names of the repository/repositories and accession number(s) can be found in the article/Supplementary Material.

## Author Contributions

RA, YK, and HK conceived and designed the research. RA, TE, EY, TW, HI, AS, and SI performed the experiments. RA, TE, and YN analyzed the data. HK, SP, YY, and YK supervised the study. RA, YK, and HK wrote the manuscript. HK, YK, YY, and MK contributed to new reagents or analytical tools. All authors have approved the manuscript.

## Funding

This work was supported by JSPS KAKENHI grant Numbers 19K05753 and 21H02088.

## Conflict of Interest

The authors declare that the research was conducted in the absence of any commercial or financial relationships that could be construed as a potential conflict of interest.

## Publisher’s Note

All claims expressed in this article are solely those of the authors and do not necessarily represent those of their affiliated organizations, or those of the publisher, the editors and the reviewers. Any product that may be evaluated in this article, or claim that may be made by its manufacturer, is not guaranteed or endorsed by the publisher.

## Footnotes

^1^ http://signal.salk.edu/tdnaprimers.2.html

^2^ https://cynin.gmi.oeaw.ac.at/

^3^ http://1001genomes.org/index.html

^4^ https://www.wessa.net/

^5^ http://www.arabidopsis.org

^6^ https://www.arabidopsis.org/tools/bulk/go/index.jsp

^7^ http://signal.salk.edu/atg1001/3.0/gebrowser.php

^8^ http://polymorph.weigelworld.org/cgi-bin/webapp.cgi

^9^ http://webs2.kazusa.or.jp/kagiana/rnr0912/indexff.html

^10^ http://atted.jp/

^11^ http://PlantPan.mbc.nctu.edu.tw

^12^ http://neomorph.salk.edu/dap_web/pages/

^13^ http://ppdb.agr.gifu-u.ac.jp/ppdb/cgi-bin/index.cgi
