# Supplementary material for: Expression GWAS of PGIP1 Identifies STOP1-Dependent and STOP1-Independent Regulation of PGIP1 in Aluminum Stress Signaling in Arabidopsis
Source: Front Plant Sci. 2021 Dec 17;12:774687. doi: 10.3389/fpls.2021.774687 (PMC8719490; doi:10.3389/fpls.2021.774687)
Supplement: Supplementary file 1 [file Image_1.pdf]

Supplementary Figure S1

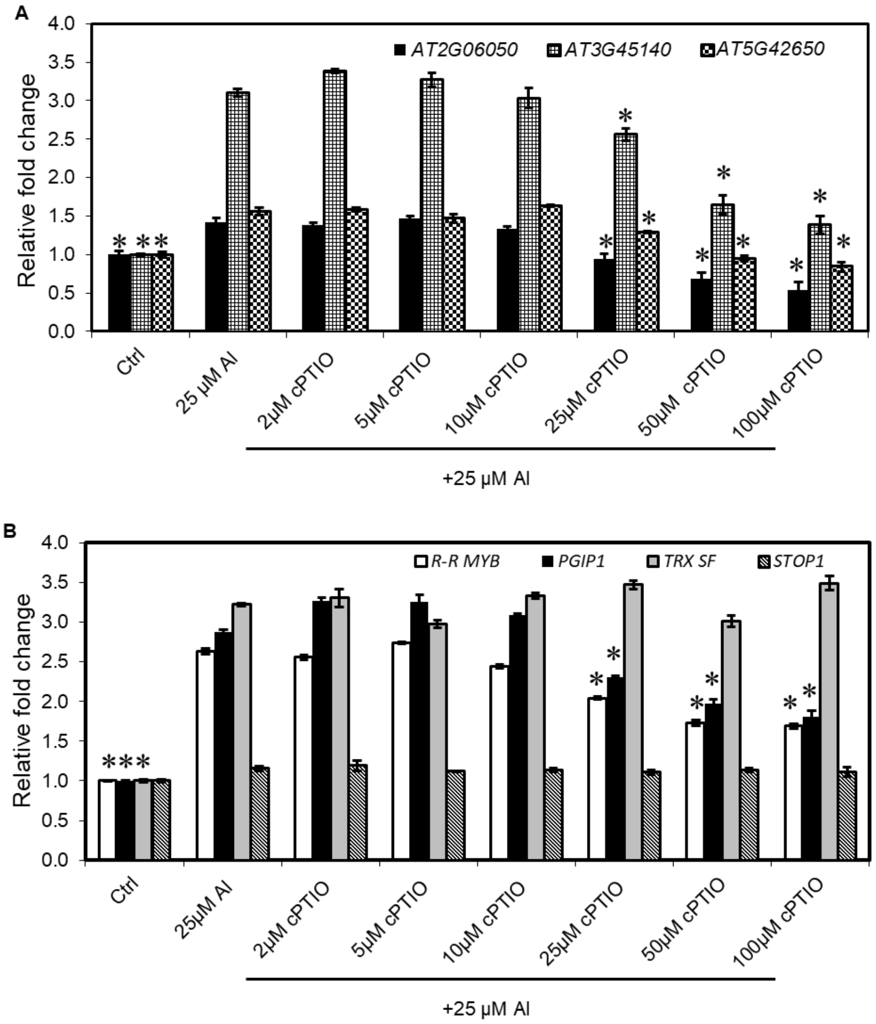

Fig. S1. Effect of NO scavenger (cPTIO) on Al stress signaling. (A) Expression analysis of NO marker genes (*AT2G06050*, *AT3G45140*, and *AT5G42650*) in the shoots of Arabidopsis under Al stress. (B) Effect of cPTIO on the expression of genes identified in eGWAS (*NAC27*, *TRX SF*, and *R-R MYB*), *PGIP1*, and *STOP1* in the shoots of Arabidopsis under Al stress. Ten-day-old seedlings were exposed to solutions containing (-Al; Ctrl), (+25 µM Al), or (+25 µM Al with different cPTIO concentrations) for 24 h. *UBQ1* was used as an internal control. Average values of three biological replicates are presented with standard errors. Relative fold changes from the control (-Al) are shown. An asterisk indicates significant difference from 25 µM Al treatment (Student's *t*-test, $P < 0.05$). Primers used for qRT-PCR are listed in Table S2.

Supplementary Figure S2

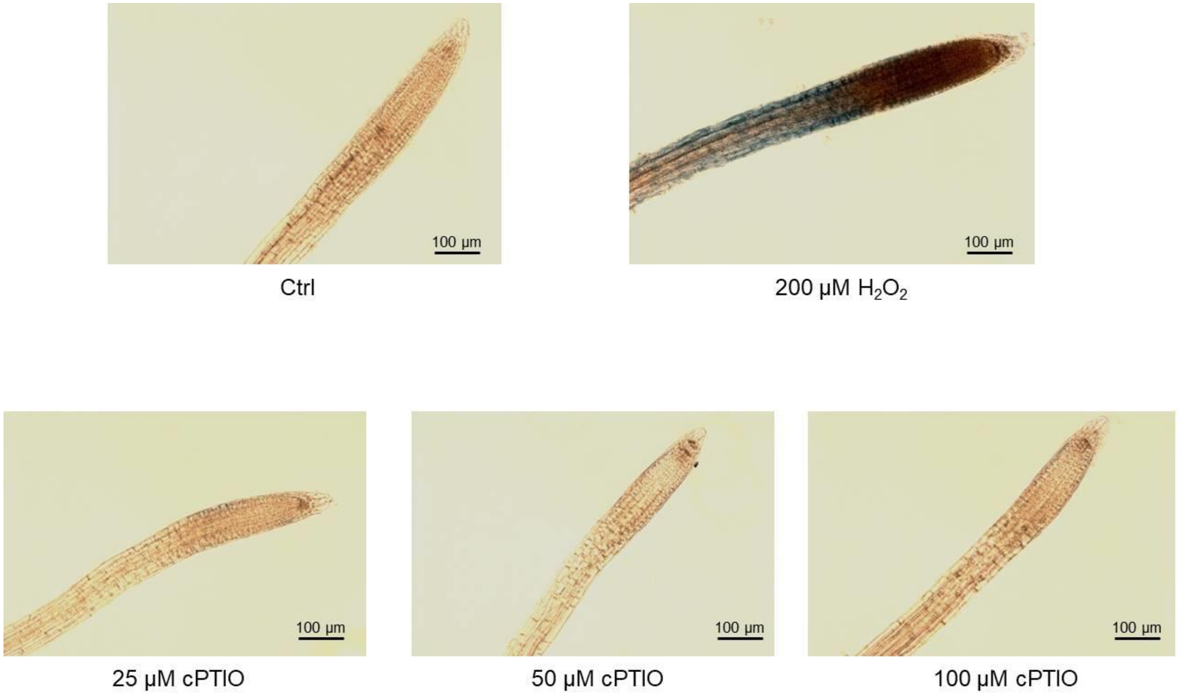

Fig. S2. Root tip viability after treatment with NO scavenger (cPTIO). Wild-type Arabidopsis seedlings were treated with 25 µM $AlCl_3$ along with 25, 50, or 100 µM cPTIO, and thereafter were stained with 0.5% Evans blue for 15 min. H_2O_2 treatment (200 µM; Sadhukhan et al., 2017) was used as a positive control. After repeated washing with water, the seedlings were mounted on glass slides and the roots were visualized with an AxioCam MRc5 microscope (Zeiss, Tokyo, Japan).

Supplementary Figure S3

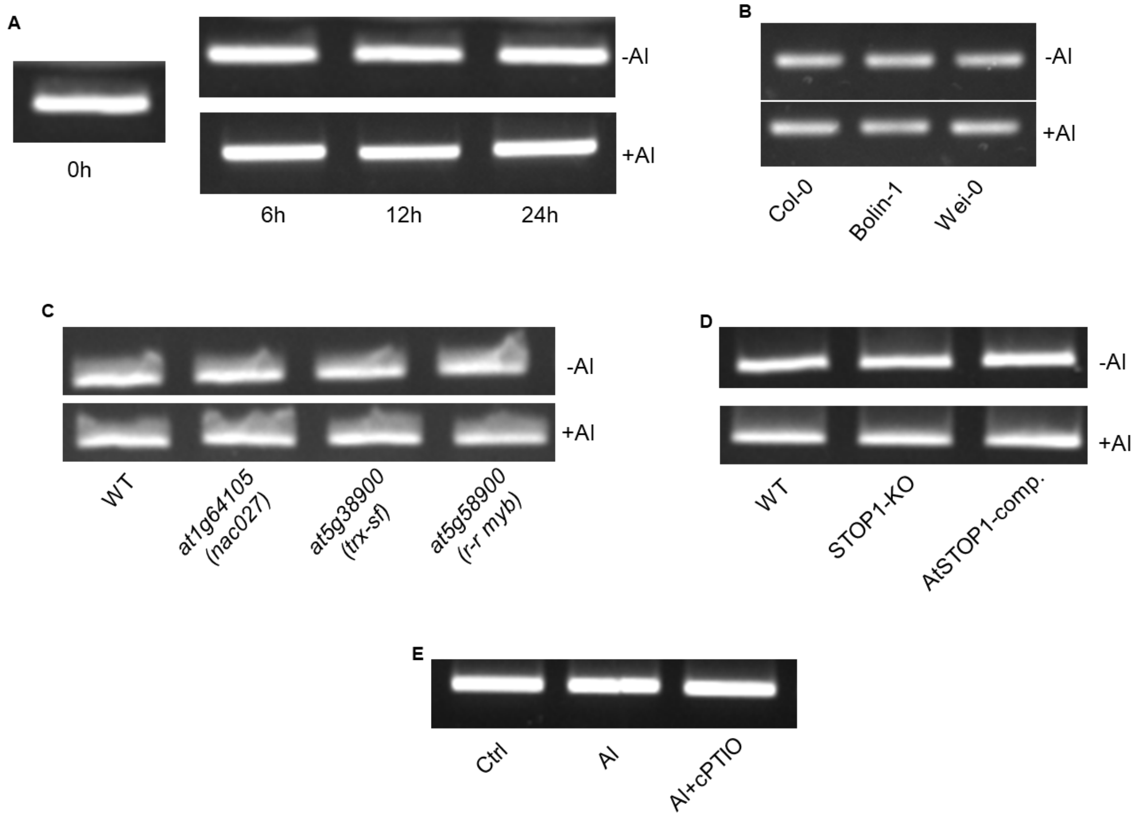

Fig. S3. Semi-quantitative PCR for expression of internal standard (*UBQ1; AT3G52590*) used in this study to normalize the gene of interest in qRT-PCR. (A) *UBQ1* expression in the shoots of Col-0 under control (–Al) or 25 µM $AlCl_3$ (+Al) treatment at different time points. (B) Expression of *UBQ1* under control (–Al) or 25 µM $AlCl_3$ (+Al) treatment for 24 h in the shoots of Col-0 along with 2 accessions i.e. Bolin-1 (shows higher expression of *PGIP1*) and Wei-0 (shows lower expression of *PGIP1*) among the 83 used in the eGWAS. (C) Expression of *UBQ1* under control and 25 µM Al treatment for 24 h in the shoot of wild-type (WT, Col-0) and T-DNA insertion mutants of genes shows significant reductions of *PGIP1* expression. (D) Expression of *UBQ1* under control (–Al) or 25 µM $AlCl_3$ (+Al) treatment for 24 h in the shoots of WT, *STOP1*-KO and AtSTOP1-complemented line. (E) Expression of *UBQ1* in the shoot of Col-0 treated with solutions containing (-Al; Ctrl), (+25 µM Al), or (+25 µM Al with 50 µM cPTIO; NO scavenger) for 24 h. PCR products were separated by 3% agarose gel electrophoresis and visualized with GelRed staining.

Supplementary Figure S4

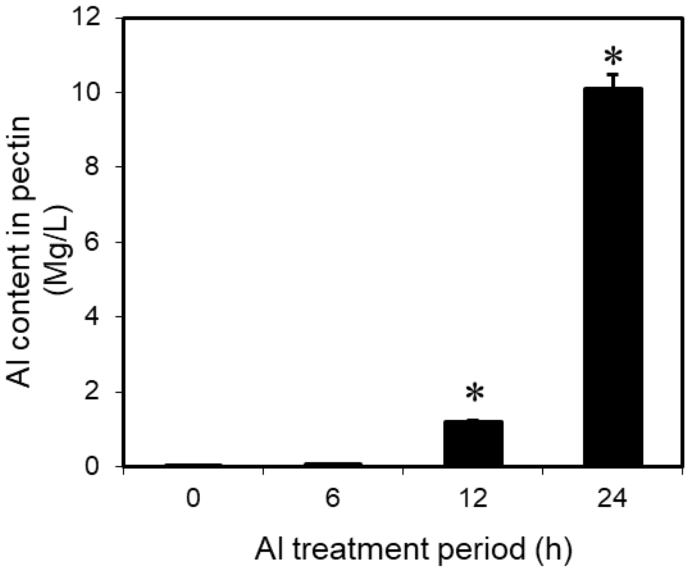

Fig. S4. Al content in the shoot cell wall pectin. Al content in pectin under Al stress at different time points of treatment are shown. The shoots were excised from 10-day-old approximately 500 seedlings treated with either 0 or 25 μM $AlCl_3$ for 0, 6, 12, and 24 h. Pectin was isolated and prepared to measure Al accumulation by ICP-MS. Average data of three replicates are presented with standard errors. An asterisk indicates significant difference from 0 h (Student's *t*-test, $P < 0.05$).

Supplementary Figure S5

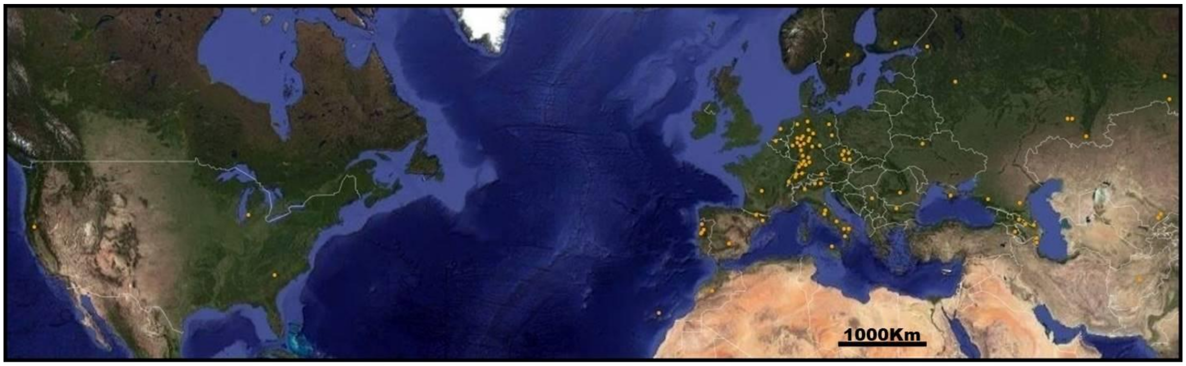

Fig. S5. Geographical distribution of *Arabidopsis thaliana* accessions used in this study. The origins of accessions are indicated as yellow dots and detailed in Table S4. Briefly, the information of accessions retrieved from 1001 Genomes database was geographically pinpointed (Atanasov et al., 2016) by using Google Earth (https://www.google.com/earth/).

Supplementary Figure S6

Locus	GO term
AT1G10540	transmembrane transporter activity
	transmembrane transport
	nucleus
	plasmodesma
	transporter activity
AT1G62050	cytoplasm
	apoplast
	negative regulation of nitrogen compound metabolic process
	negative regulation of cell cycle
	tissue development
	flower development
	molecular_function_unknown
	nucleus
	cell division
	response to light stimulus
	cell differentiation
	meiotic cell cycle process
AT1G64105	DNA binding
	DNA-binding transcription factor activity
	regulation of transcription, DNA-templated
	nucleus
AT2G18880	vernalization response
	cellular response to cold
	response to cold
	regulation of gene expression, epigenetic
AT2G21850	intracellular signal transduction
	cell differentiation
	root development
	tissue development
	nucleus
AT2G21950	protein ubiquitination
	organelle organization
	chloroplast
AT3G24480	root development
	defense response
	cell differentiation
	plant epidermis development
	plant-type cell wall
	lipid metabolic process
	organic hydroxy compound metabolic process
	extracellular region
	structural constituent of cell wall
	phyllome development
	plasmodesma
	developmental growth
	plant-type cell wall organization or biogenesis
	cell wall biogenesis

Locus	GO term
AT5G15300	chloroplast RNA modification
	chloroplast
	nucleus
AT5G26300	anchored component of membrane
	extracellular region
	anchored component of membrane
AT5G26330	electron transfer activity
	anchored component of membrane
	anchored component of plasma membrane
AT5G37500	ion transmembrane transport
	response to jasmonic acid
	nucleus
	ion transport
	response to cold
	response to water deprivation
	outward rectifier potassium channel activity
	voltage-gated potassium channel activity
	response to calcium ion
	ion transmembrane transporter activity
	response to abscisic acid
	protein binding
AT5G38860	protein binding
	protein dimerization activity
	regulation of transcription, DNA-templated
	nucleus
	DNA-binding transcription factor activity
AT5G38900	chloroplast
	oxidoreductase activity
AT5G43460	response to lipid
	chloroplast
	molecular_function_unknown
	hormone-mediated signaling pathway
	endoplasmic reticulum
AT5G50940	nucleus
	cytoplasm
	regulation of gene expression
	mRNA binding
	transcription by RNA polymerase II
AT5G58900	nucleus
	carbohydrate derivative metabolic process
	small molecule metabolic process
	regulation of transcription, DNA-templated
	response to red light
	DNA-binding transcription factor activity
	transcription cis-regulatory region binding
AT5G58910	hydroquinone:oxygen oxidoreductase activity
	extracellular region
	copper ion binding
	secondary metabolite biosynthetic process
	oxidoreductase activity

Fig. S6. GO term for the genes in which suggestive SNPs were located from GWAS of *PGIP1* expression. GO annotations of the genes in Table 1 based on Gene Ontology at TAIR. GO terms that may be involved in the regulation of gene expression used for candidate gene selection are shown in red.

Supplementary Figure S7

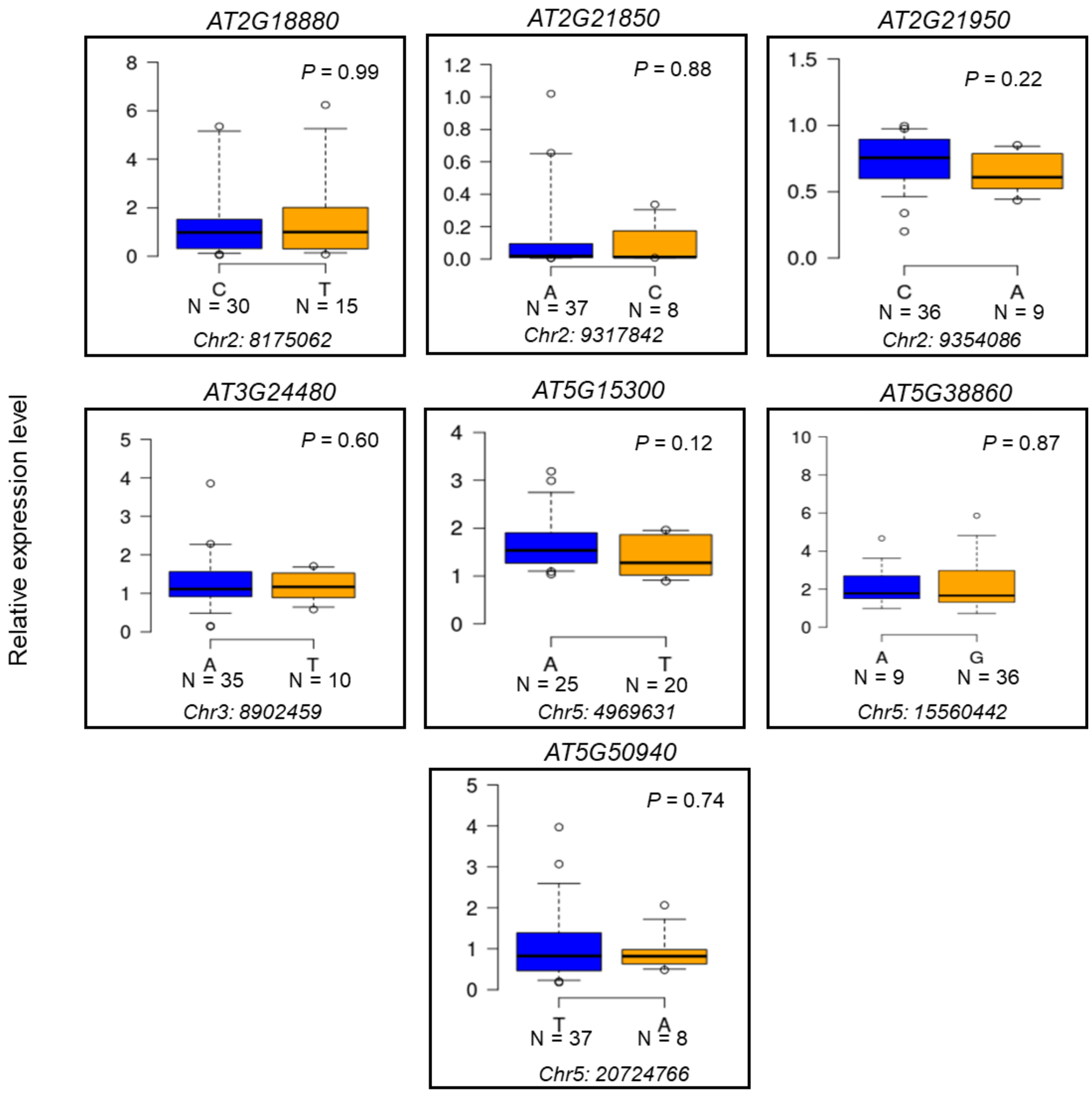

Fig. S7. Expression level polymorphisms of the candidate genes (Table 1) identified by eGWAS. Expression of these genes was monitored in 45 randomly chosen accessions among the 83 used in the eGWAS. Accessions are grouped according to SNP alleles at particular physical chromosome positions indicated below each box plot. Number of accessions of each SNP allele is shown under each SNP. Expression levels of each gene are relative to Col-0. *UBQ1* was used as the internal control. Significant difference between the average values of group occurred at $P < 0.05$ (Student's *t*-test). Primers used for qRT-PCR are listed in Table S2.

Supplementary Figure S8

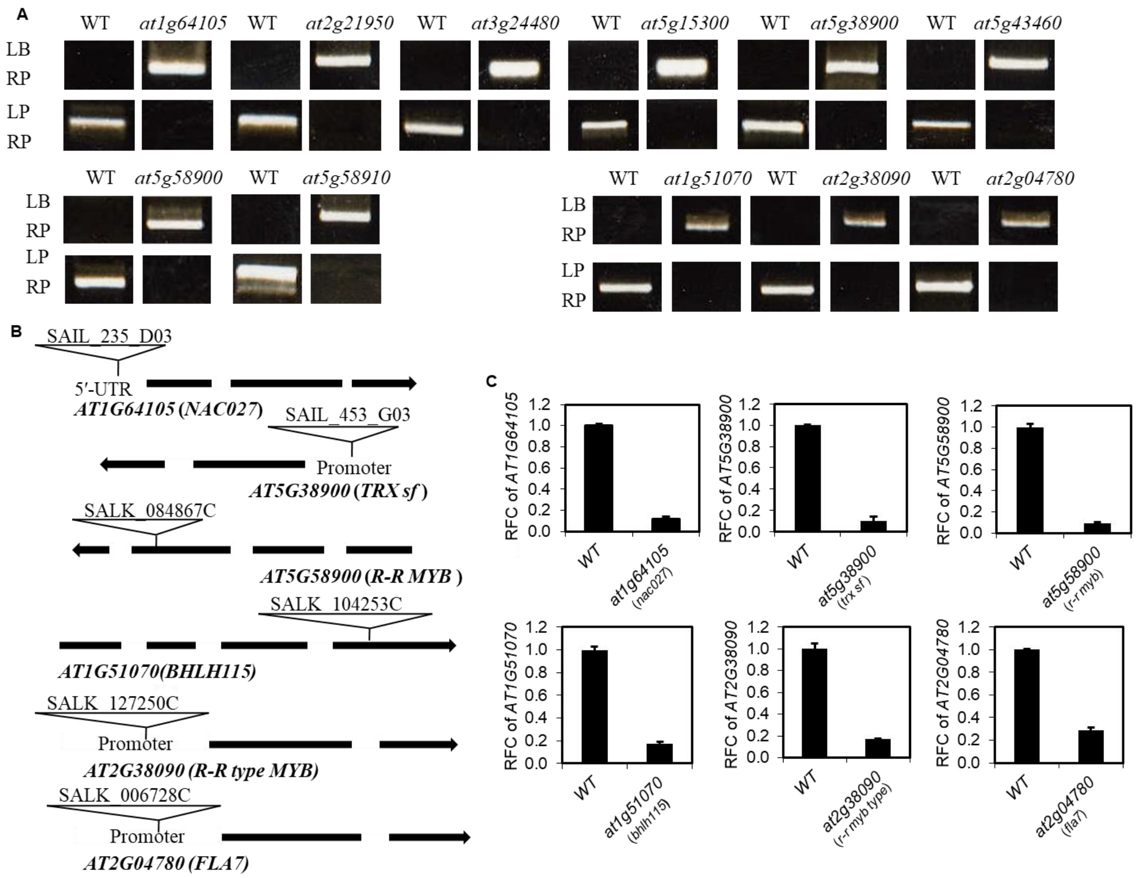

Fig. S8. Validation of homozygous T-DNA insertion mutants. (A) T-DNA insertion mutants used in the study were tested for homozygosity using PCR-based method as recommended by SALK T-DNA express. Primers used to check genomic integration of T-DNA are listed in Table S1. RP; reverse genomic primer; LP; forward genomic primer, LB; T-DNA left border primer as recommended by SALK T-DNA express. (B) Positions of insertion of T-DNA in the mutated gene that regulates *PGIP1* expression. Solid lines indicate exon regions of each gene. (C) Expression levels of respective genes regulating *PGIP1* were further checked in the T-DNA insertion knockouts or knockdown mutants. Expression levels are expressed as relative fold changes (RFC) compared to WT. *UBQ1* was used as the internal control. Primers used for qRT-PCR are listed in Table S2.

Supplementary Figure S9

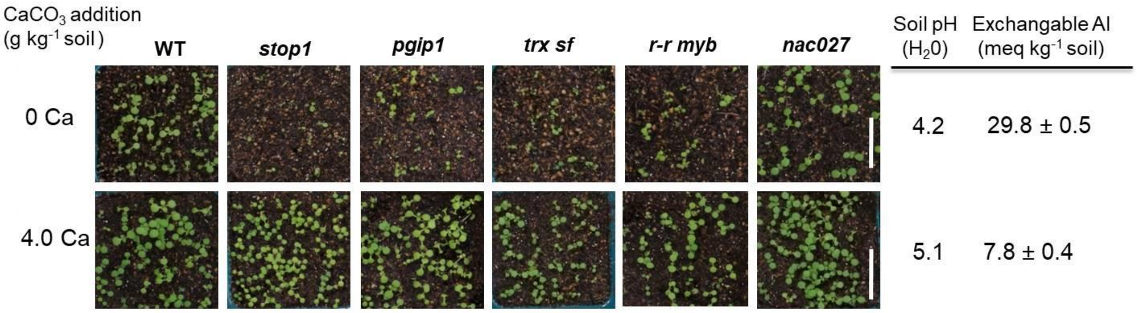

Fig. S9. Growth of *Arabidopsis* (Col-0; WT) and T-DNA insertion mutants of the genes regulating *PGIP1* in acidic soil containing exchangeable aluminum. One hundred seeds were sown in acidic soil with different levels of liming (0 and 4.0 g $CaCO_3$ kg^{-1} soil). Figure shows the growth at two weeks and chemical properties of the soil. White bar indicates 1 cm.

Supplementary Figure S10

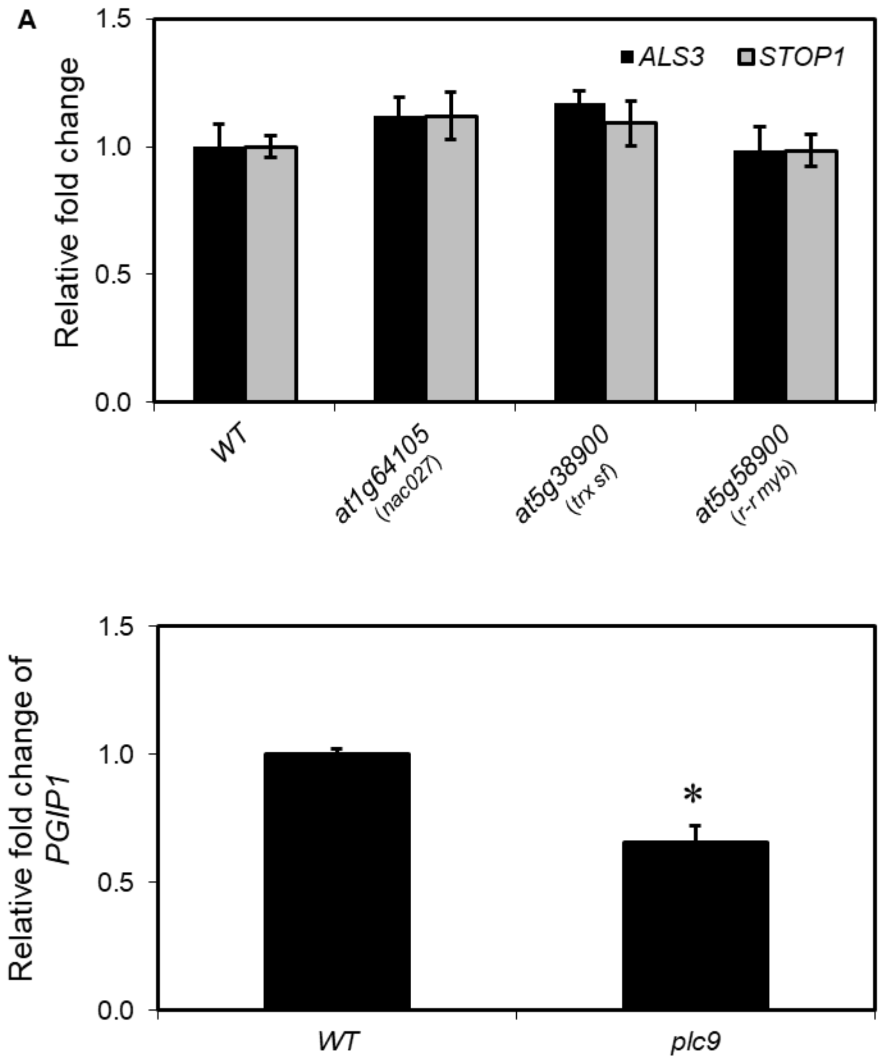

Fig. S10. Relationship between STOP1 and the genes detected by eGWAS that regulate the expression of *PGIP1* under Al stress. (A) *ALS3* and *STOP1* expression in the mutants of genes identified by eGWAS for *PGIP1* expression in shoots under Al stress. (B) *PGIP1* expression in the *plc9* (knockout of *PLC9;* Sadhukhan et al., 2020) shoots under Al stress. Ten-day-old seedlings were exposed to 25 µM Al for 24 h. *UBQ1* was used as an internal control. Average values of three biological replicates are presented with standard errors. Gene expressions levels are relative to WT. Significant reductions in RFC from the Al-treated WT sample are indicated by asterisks (Student's *t*-test, $P < 0.05$). Primers used for qRT-PCR are listed in Table S2.

Supplementary Figure S11

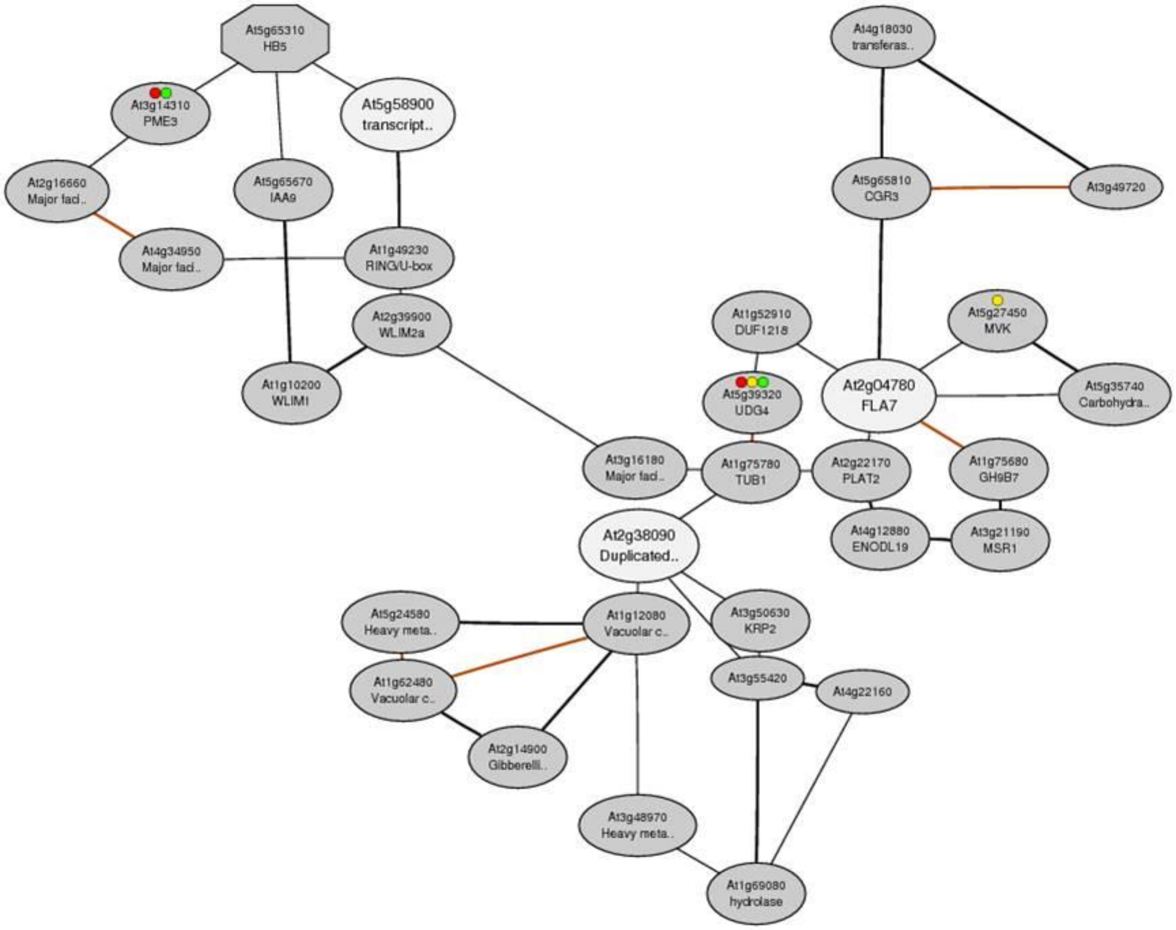

Fig. S11. Co-expression network containing the candidate genes that regulate *PGIP1* detected by eGWAS and RNR database. The co-expression network was constructed by ATTED-II using six genes regulating *PGIP1* (*TRX SF*, *NAC027*, *R-R MYB*, *BHLH115*, *AT2G38090*, and *FLA7*) as query genes. White and gray ellipses indicate query genes and added co-expressed genes, respectively. Colored circles indicate the genes involved in enriched biological processes obtained from Kyoto Encyclopedia of Genes and Genomes (KEGG; https://www.genome.jp/kegg/), such as pentose and glucuronate interconversions (red; KEGG ID: ath00040), metabolic pathway (green; KEGG ID: ath01100), and amino sugar and nucleotide sugar metabolism (yellow; KEGG ID: ath00520)

Supplementary Figure S12

#	Accession	-1031	-1023	-1010	-690	-546	-211	1)-61, 2)-66, 3)-59	-18	+29*	+45	NAC027 Expression	PGIP1 Expression
**1)**	**Ciste-1**	C	T	T	G	G	**T**	**36bp Deletion**	2bp Deletion	**C**	V	**2.1**	**0.76**
**2)**	**TueWa1-2**	C	T	T	G	A	**T**	**25bp Deletion**	2bp Deletion	**C**	V	**1.4**	**0.30**
**3)**	**Rsch-4**	C	T	T	T	G	**T**	**45bp Deletion**	AA	**C**	L	**2.2**	**0.86**
4)	Xan-1	C	T	T	T	A	A		2bp Deletion	S	L	1.0	0.13
5)	Vie-0	T	A	A	G	G	A		AA	S	V	0.8	0.14
6)	El-0	T	A	A	G	G	A		AA	S	V	0.7	0.09

Fig. S12. Promoter region sequence and amino acid polymorphism of the *NAC027*. Approximately 1000 bp upstream from the start codon (indicated by minus) and the exon (indicated by plus) were compared for accessions showing high and low expression of the *NAC027*. Bold indicates polymorphisms associated with high *NAC027* expression and their accessions. The asterisk indicates the position of the SNP detected by GWAS in this study.

Supplementary Figure S13

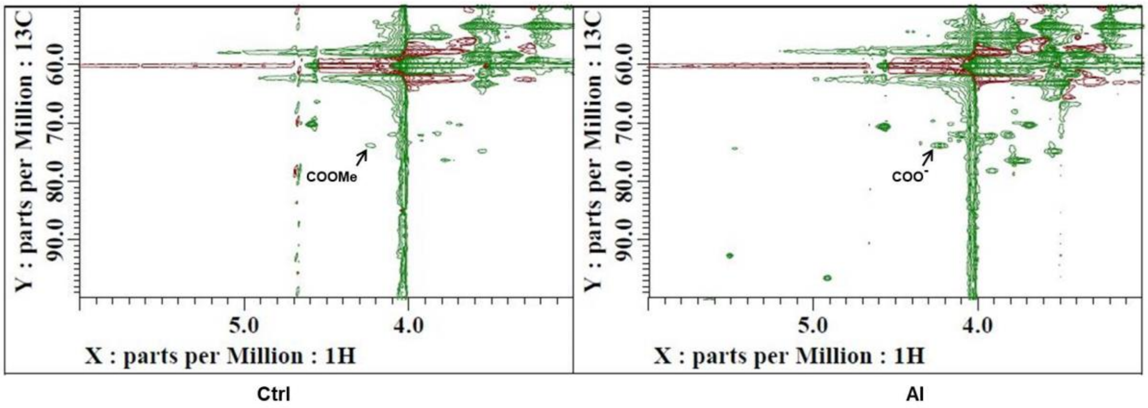

Fig. S13. HSQC (heteronuclear single quantum coherence) two dimensional NMR analysis of pectin (CDTA extract) from *Arabidopsis* shoots after exposure of the roots to control (−Al) or Al rhizotoxic (25 µM $AlCl_3$) solutions for 24 h. HSQC spectrum showing pectin methylesterification and demethylesterification under Ctrl and Al stress.

Table S1. List of oligonucleotides used to validate T-DNA insertion mutants used in this study

		Sequence (5' to 3')	
**AGI**	**SALK No.**	**Left Primer (LP) Sequence**	**Right Primer (RP) Sequence**
AT1G64105	SAIL_235_D03	TTGAATCATCAGAAAGCAAAGAG	TTATTTGCTTTCAGTCCACCG
AT2G21950	GABI_707C08	ACCAAAATCCTTTCAAAACCG	AGAGCCCTTTTGTGAACAACC
AT3G24480	GABI_017A08	CAAATCGGTTCGATAGCAAAC	CATTTCCATGGTTGTATTCCG
AT5G15300	SALK_044494C	GTCCTACCACTAACCCCGTTC	CTCCACTCAAGCTTCGAACAC
AT5G38900	SAIL_453_G03	AATAGGAAAACGCATCAAACG	TTCCTACAAAGCACCATGGAC
AT5G43460	SALK_067877C	TCTGTGATTGATAGCGACGTG	ATCGGAAAACAATCCGAAATC
AT5G58900	SALK_084867	TTGCCGGATGAAGTACTTTTG	TTCGGACATGGAGGTTATGAG
AT5G58910	SALK_064093C	GAGTTTTTCGGGACTTCCAAG	CAGTAAAGCTCGGCGTTAATG
AT1G51070	SALK_104253C	AAGACTTGGGATTTTTGATTGG	AAGCCATCCACTTCAACACTG
AT2G38090	SALK_127250C	CAACATTGTTTTCTATAGCATCG	TTTCTTCAGCTGTCCATTTGG
AT2G04780	SALK_113729C	TTATAATGGTTGCCATTTGCC	AATCTCCATGGTTATTTCCGG

Table S2. List of oligonucleotides used in this study for qRT-PCR

	Sequence (5' to 3')	
**AGI**	**Forward Primer Sequence**	**Reverse Primer Sequence**
AT1G64105	GGGTGAGGAAATTGGTGGAA	ACGTCCATTGAAGACTCTCCTG
AT2G21950	CTTAATGCCCTGAGCCAAGA	CAAAGAAATGCACATGCAGAAG
AT2G21850	TCTGAGGTTATGCCTCTTTGCTT	AAAACAGTGGCACAGGCACTAC
AT2G18880	GCATCCTGAGATTGGTGGTG	CAATGCTTCTCATCAGATCTCCTCT
AT3G24480	CGCCACCATCAATCCATTAC	ATGGCCCTTCAAATTCAGGT
AT5G15300	AACGCTTCATTGGCGAACTA	CTTCCTATAAAATGTGGTGCTCAGA
AT5G38860	CATTCCCAGAAGAGAATAAGACGA	CCTGACTCACCGAAAGTAACAAA
AT5G38900	GGAGATTCTCTGTTTCTGTTGTTTG	GCAAGAAGAAGAAGAGTGCTTGA
AT5G43460	CTGTGATTTGATATTGGGGATCTTG	GGTCATGAAATCATTGTTCTGTAGC
AT5G50940	TCTCAGAAAGATTGTGGAGCTCTT	CCTTGGAGAGGTTGCAGCTA
AT5G58900	AGTGGCGTTCATTCATACGG	CATTGTGTTTTGGGCGTCA
AT5G58910	GGTGGTTGGACTGCCATAAGA	GTGGAGGAAGCAATGACTGG
AT1G51070	GCTTAAAGCTGCTGCTTCTCTCT	TGCAGTGGACTATGTCACCAA
AT2G38090	ACAAGGTGAAGATCTCGGAGAAG	ATATGCACGCCGATTAAGAGAA
AT2G04780	CTCCGGACAAAAGCTGCTAC	CTCAATCTGCAAAACCATCTGA
At5g42650	GCTTTTAGGAGCCAAGGGTAAA	CGAACATGTAGAGCAGCAACAG
AT3G45140	GGCAAGCTCCAATATCTAGAAGGA	GAACACCCATTCCGGTAACA
At2g06050	GCGGTTCAAGATTGATGGAGA	CAAACTCAGAGGCGGGAAAA
PGIP1	TAAACCAAGCTTATCTCTAGGATTA	CCATCAAATAAAACATTTTGAAAATGTGC
ALS3	TATCGATCCTTGCCGGGACTTCA	GCTTGTCTTGGCGTTGCTCCTA
UBQ1	TCGTAAGTACAATCAGGATAAGATG	CACTGAAACAAGAAAAACAAACCCT
STOP1	CCTGGAATGACTGATGGGAGGA	TCCTGGGCGAGAAACCACA

Table S3. List of probe sequences used for DNA-protein binding assay

**Probe name**	**Probe Sequence (5' to 3')**
AT5G06860 (PGIP1)-STOP1bind-FW	TATAACTTGCACCGGTTATAGCCTAATTAT
AT5G06860 (PGIP1)-STOP1bind-Rv	ATAATTAGGCTATAACCGGTGCAAGTTATA
AT5G38900 (TRX SF)-STOP1bind-FW	ACCGTCTAGATACGACCTACCGATTTTTTG
AT5G38900 (TRX SF)-STOP1bind-RV	CAAAAAATCGGTAGGTCGTATCTAGACGGT
AT5G06860 (PGIP1)-mutated-FW	ATAATTAGGCTATAAAAAAAAAAAGTTATA
AT5G06860 (PGIP1)-mutated-Rv	TATAACTTTTTTTTTTTATAGCCTAATTAT
AT5G38900 (TRX SF)-mutated-FW	ACCGTCTAGATTTTTTTTTTTGATTTTTTG
AT5G38900 (TRX SF)-mutated-RV	CAAAAAATCAAAAAAAAAAATCTAGACGGT

Table S4. Expression levels of *PGIP1* in *Arabidopsis thaliana* accessions used in this study

**Accession name**	**Fold change in *PGIP1* (-Log$_2$)**	**Location**	**Country**
Go-0	-0.099	Goettingen	Germany
Fr-4	-0.193	Frankfurt	Germany
Rsch-4	-0.223	Rschew/Starize	Russia
Ei-2	-0.285	Eifel	Germany
Ciste-1	-0.400	Cisterna de Latina	Italy
Voeran-1	-0.492	Voeran, South Tyrolia	Italy
Copac-1	-0.548	Copac	Serbia
Db-0	-0.550	Dombachtal/Ts.	Germany
Nd-0	-0.680	Niederzenz	Germany
Hn-0	-0.725	Hennetalsperre	Germany
Ey15-2	-0.834	Eyach	Germany
Can-0	-0.873	Canary Islands	Spain
Koch-1	-0.897	Zhytomir Oblast; Radomyshlsky	Ukraine
An-1	-0.902	Antwerpen	Belgium
In-0	-0.930	Innsbruck	Austria
Ag-0	-1.044	Argentat	France
Apost-1	-1.077	Sant Piedro Apostolo	Italy
Bu-0	-1.099	Burghaun/Rhon	Germany
Bay-0	-1.111	Bayreuth	Germany
Timpo-1	-1.114	Timpo Ulivi, Cosenza	Italy
Co-2	-1.136	Coimbra	Portugal
Angel-1	-1.136	Sant Angelo	Italy
Fei-0	-1.175	Santa Maria da Feira village	Portugal
Blh-2	-1.214	Bulhary	Czech Republic
En-1	-1.245	Enkheim/Frankfurt	Germany
Dra-2	-1.248	Drahonin	Czech Republic
Br-0	-1.333	Brunn	Czech Republic
TueWa1-2	-1.333	Tubingen - Wanne	Germany
Aitba-2	-1.336	Ait Barka, Marrakech	Morocco
Krazo-2	-1.336	Krasnaja	Russia
Yeg-1	-1.381	Yeghegis	Armenia
Lag2.2	-1.400	Lagodechi	Georgia
Dra-1	-1.427	Drahonin	Czech Republic
Pla-0	-1.458	Playa de Aro	Spain
Dr-0	-1.470	Dresden	Germany
Pna-10	-1.486	Benton Harbor, Midwest	USA
Galdo-1	-1.502	Galdo	Italy
Chi-1	-1.604	Chisdra	Russia
Nok-3	-1.617	Noordwijk	Netherlands
Fi-1	-1.662	Frickhofen	Germany
Bsch-2	-1.737	Buchschlag/Frankfurt am Main	Germany
Jablo-1	-1.806	Jablokovec	Bulgaria
Kastel-1	-1.806	Republic of Crimea	Ukraine
Bd-0	-1.831	Berlin/Dahlem	Germany
HKT2.4	-1.831	Heiligkreuztal	Germany

Shigu-1	-1.842	Aleksandrovka	Russia
Hs-0	-1.847	Hannover/Stroehen	Germany
Shigu-2	-1.878	Aleksandrovka	Russia
Istisu-1	-1.921	Istisu	Azerbaijan
Es-0	-1.927	Espoo	Finland
Bs-1	-1.932	Basel	Switzerland
Sij2	-1.938	Sidzhak	Uzbekistan
Sha	-1.943	Shirdagh	Afghanistan
Koz2	-1.983	Altaijskij Kraj	Russia
Co-3	-2.071	Coimbra	Portugal
Bs-2	-2.077	Basel	Switzerland
Ws-0	-2.114	Wassilewskija	Russia
Ciste-2	-2.171	Cisterna de Latina	Italy
Vash-1	-2.171	Vashlovani Reserve	Georgia
Kidr-1	-2.178	Kidrjasovo, Orenburgskaja Oblast	Russia
Rue3-1-31	-2.198	Rubgarten	Germany
Tuescha9	-2.238	Tübingen - Schaal	Germany
Wei-0	-2.279	Weiningen	Switzerland
Kly4	-2.286	Altajski kraj	Russia
TueV13	-2.322	Tubingen - Volksbank	Germany
Ge-0	-2.427	Geneva	Switzerland
Buckhorn Pass	-2.565	Buckhorn Pass	USA
Omo-2-3	-2.626	Ostra	Sweden
Bor-4	-2.644	Borky	Czech Republic
Sij1	-2.727	Sidzhak	Uzbekistan
Se-0	-2.747	San Eleno	Spain
Xan-1	-2.796	Xanbulan	Azerbaijan
Mz-0	-2.816	Merzhausen/Ts.	Germany
Pa-1	-2.837	Palermo	Italy
Vie-0	-3.000	Near Vielha	Spain
Rovero-1	-3.083	Rovero d. Luna	Italy
Valsi-1	-3.211	Valsinnica	Italy
Gie-0	-3.366	Gieben	Germany
El-0	-3.458	Ellershausen	Germany
Lerik1-3	-5.158	Lankaran and Lerik	Azerbaijan
Star-8	-8.381	Starzach	Germany
Col-0	0.000	Columbia	USA
Bolin-1	0.034	Bolintin Vale	Romania
